# Identifying the potential miRNA biomarkers based on multi-view networks and reinforcement learning for diseases

**DOI:** 10.1093/bib/bbad427

**Published:** 2023-11-28

**Authors:** Benzhe Su, Weiwei Wang, Xiaohui Lin, Shenglan Liu, Xin Huang

**Affiliations:** School of Computer Science and Technology, Dalian University of Technology, Dalian 116024, Liaoning, China; School of Computer Science and Technology, Dalian University of Technology, Dalian 116024, Liaoning, China; School of Computer Science and Technology, Dalian University of Technology, Dalian 116024, Liaoning, China; School of Innovation and Entrepreneurship, Dalian University of Technology, Dalian 116024, Liaoning, China; School of Mathematics and Information Science, Anshan Normal University, Anshan 114007, Liaoning, China

**Keywords:** miRNA data analysis, miRNA–disease relations, reinforcement learning, biomarker identification

## Abstract

MicroRNAs (miRNAs) play important roles in the occurrence and development of diseases. However, it is still challenging to identify the effective miRNA biomarkers for improving the disease diagnosis and prognosis. In this study, we proposed the miRNA data analysis method based on multi-view miRNA networks and reinforcement learning, miRMarker, to define the potential miRNA disease biomarkers. miRMarker constructs the cooperative regulation network and functional similarity network based on the expression data and known miRNA–disease relations, respectively. The cooperative regulation of miRNAs was evaluated by measuring the changes of relative expression. Natural language processing was introduced for calculating the miRNA functional similarity. Then, miRMarker integrates the multi-view miRNA networks and defines the informative miRNA modules through a reinforcement learning strategy. We compared miRMarker with eight efficient data analysis methods on nine transcriptomics datasets to show its superiority in disease sample discrimination. The comparison results suggested that miRMarker outperformed other data analysis methods in receiver operating characteristic analysis. Furthermore, the defined miRNA modules of miRMarker on colorectal cancer data not only show the excellent performance of cancer sample discrimination but also play significant roles in the cancer-related pathway disturbances. The experimental results indicate that miRMarker can build the robust miRNA interaction network by integrating the multi-view networks. Besides, exploring the miRNA interaction network using reinforcement learning favors defining the important miRNA modules. In summary, miRMarker can be a hopeful tool in biomarker identification for human diseases.

## INTRODUCTION

MiRNAs are small endogenous non-coding RNAs regulating the gene expression at the posttranscriptional level [1]. Studies have shown that miRNAs play crucial roles in many important biological processes, including cell differentiation and proliferation [2], tumorigenesis and metastasis [3]. It is helpful to explore the close associations between miRNAs and human diseases for understanding the disease pathogenesis. Moreover, miRNAs show many advantages as the potential disease biomarkers, such as excellent tissue specificity, high reliability of condition identification and the favorable stability in body fluids [4]. Hence, identifying the miRNA biomarkers related to the disease occurrence and development is of great significance for disease diagnosis and prognosis.

Biological network analysis provides the strong support for understanding the changes of biomolecular interactions during the disease development as well as defining the potential biomarkers [5]. Common network analysis methods are based on the molecular correlation. Zhang *et al*. [6] proposed the weighted gene co-expression network analysis (WGCNA) to explore the co-expression patterns of genes based on the weighted correlation network. It defines the dense modules by a weighted topological overlap measure. WGCNA has been adopted in the disease studies for other different omics, such as transcriptomics [7–9]. The dynamic network biomarker (DNB) method evaluates the dynamic changes of molecular correlation and the SD of the molecules to define the potential disease biomarkers [10, 11]. The DNB can highlight the critical tipping points in the disease progression. These network-based analysis methods provide reference for miRNA network construction based on expression data.

In addition to the miRNA expression data, various miRNA-related knowledge bases strongly support the understanding of the miRNA functional interactions [12]. Those miRNAs having many common regulated target genes or diseases may function similarly. Some methods evaluate the miRNA interactions by considering the experimentally validated miRNA–target relations [13–15]. The intuitive way for evaluating the miRNA functional interactions is calculating the proportion of the common targets by Jaccard index [16]. However, it discards a lot of similarity information. Some methods try to adopt the Gene Ontology (GO) annotations or protein–protein interactions (PPIs) as the additional information of target genes for inferring the miRNA interactions [17–19]. Unfortunately, the GO database has the limited annotation information for massive target genes, adverse to evaluate the miRNA interactions accurately [16]. Meanwhile, there are lots of false positives without the experimental validity in PPI databases, which may introduce the bias for calculating the miRNA functional similarity. The miRNA–disease relations [20–22] provide the direct evidence of miRNAs participating in the occurrence and development of diseases, becoming the strong choice for evaluating the miRNA functional interactions.

One miRNA usually regulates the development processes of multiple diseases, and one disease is associated with many miRNAs. The key of disease-based miRNA functional similarity calculation is assessing the disease semantic similarity accurately. Wang *et al*. [23] proposed a graph-based method to infer the disease semantic similarity based on the disease directed acyclic graphs (DAGs) from the public database Medical Subject Headings (MeSH, https://www.ncbi.nlm.nih.gov/mesh). The diseases had high semantic similarity if they had large common parts in their DAGs. Nevertheless, the DAG-based disease similarity ignores the specific disease semantic significance by equally evaluating the importance of diseases with the same distance to root disease [24]. Therefore, to obtain the miRNA functional interactions with high quality, the disease semantic information in the miRNA–disease relations should be rationally assessed and fully used.

What’s more, it is conducive to construct the robust miRNA interaction network by using the miRNA expression data and the information in knowledge bases simultaneously. The miRNA expression data contain the change information of miRNA cooperative regulation between different sample groups. And the known miRNA–disease relations sustain the assessment of miRNA functional similarity strongly. Thus, integrating the miRNA cooperative regulation and miRNA functional similarity can promote the downstream network analysis tasks, including the potential biomarker identification for diseases.

Defining the informative subnetworks based on molecular interaction networks is an important topic in biological network analysis [25]. Some methods adopt the clustering techniques to divide the biological networks [6, 11]. In general, a predefined cluster number is needed, but it is difficult to determine. Some methods adopt the heuristic strategy for identifying the important modules. Zhang *et al*. [26] proposed the network-based game theory method (NGTM) to identify the potential cancer subnetwork biomarkers by evaluating the feature contribution using the cooperative game theoretic metrics (Shapley values) in the heuristic-based module extension. But in the heuristic-based module identification methods, the heuristic information used is rather limited, making it easy to obtain local optimal results. Different from supervised and unsupervised learning, reinforcement learning (RL) aims to make decisions maximizing the long-term returns [27]. Accordingly, the RL strategy may bring more possibilities for module biomarker identification by fully exploring the solution space to obtain global optimal results. Paim *et al*. [28] tried to employ RL to detect the communities in complex networks and proposed the Q-Learning [29] for community detection (QLCD) method. Each node in the network acts as an agent, which selects one node from its nearest neighbor nodes (action space) to compose the clusters. The agent nodes learn the action policy to maximize the network modularity. However, QLCD may fail to find the competitive modules in disease studies due to its inherent simple action space and inadequate learning strategy. It is necessary to further explore the potential of reinforcement learning in defining the network biomarkers for diseases.

For identifying the potential miRNA disease biomarkers effectively, we proposed the miRNA data analysis method based on multi-view networks and reinforcement learning, miRMarker. It constructs the miRNA cooperative regulation network based on expression data. The miRNA functional similarity network is built using the known miRNA–disease relations from public knowledge bases. Then, miRMarker integrates the two miRNA networks and defines the crucial miRNA modules by a reinforcement learning strategy. We verified the effectiveness of miRMarker in disease sample discrimination by comparing it with eight effective data analysis methods on nine transcriptomics datasets. In addition, we examined the potential miRNA module biomarkers defined by miRMarker for colorectal cancer. The experimental results demonstrate the great potentials of miRMarker in defining the important module biomarkers for the diagnosis and prognosis of diseases.

## MATERIALS AND METHODS

**Figure 1:**
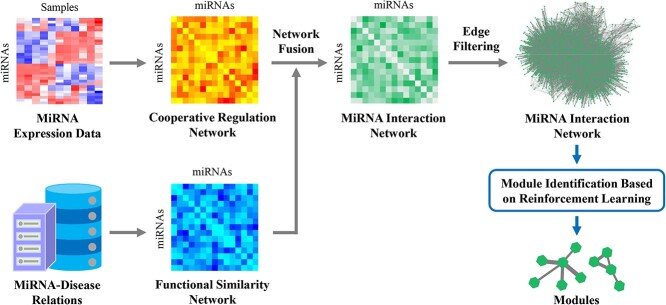
Workflow of miRMarker.

miRMarker consists of two main parts: (i) constructing the miRNA networks based on miRNA expression data and miRNA–disease relations, respectively, integrating the two networks and (ii) defining the crucial miRNA modules by a reinforcement learning strategy. Figure 1 shows the workflow of miRMarker.

### MiRNA expression datasets

In this study, nine miRNA expression datasets were collected to evaluate the effectiveness of miRMarker. All the datasets were derived from the public data repository Gene Expression Omnibus (GEO, https://www.ncbi.nlm.nih.gov/geo/), involving multiple diseases, such as hepatocellular carcinoma, thrombocytosis and colorectal cancer. Table 1 gives the details of the nine miRNA expression datasets. Datasets GSE41574, GSE67139, GSE32273, GSE34496, GSE41282 and GSE108153 are two-class datasets. Datasets GSE31164, GSE39046 and GSE35834 are multi-class datasets. We mapped all human mature miRNA names to the standard miRNA accession numbers using miRBase v22.0 [30] (see more details in Supplementary Materials available online at http://bib.oxfordjournals.org/). The expression values of the probes representing the same miRNA were averaged.

**Table 1:** Details of miRNA expression datasets

Datasets	Involved diseases	Number of features	Number of samples	Number of classes
GSE31164	Hepatocellular carcinoma	818	110 (30:60:20)	3
GSE39046	Thrombocytosis	372	79 (30:27:22)	3
GSE41574	Sickle cell disease	818	43 (17:26)	2
GSE67139	Hepatocellular carcinoma	812	115 (57:58)	2
GSE32273	Ulcerative colitis	812	132 (66:66)	2
GSE34496	Head and neck squamous cell carcinomas	812	69 (25:44)	2
GSE35834	Colorectal cancer	812	78 (23:31:24)	3
GSE41282	Renal cell cancer	812	38 (18:20)	2
GSE108153	Colorectal cancer	1972	42 (21:21)	2

### MiRNA–disease relations

The manually curated miRNA–disease relations were extracted from the two largest knowledge bases miRCancer [20] and miR2Disease [21]. miRCancer uses the text mining technique to extract the miRNA–cancer associations from the medical literatures in the PubMed database (https://pubmed.ncbi.nlm.nih.gov/), and then the associations are revised manually. miR2Disease provides the comprehensive regulatory associations between miRNAs and human diseases curated from the published papers. We downloaded the latest versions of miRCancer (9080 entries, downloaded in June 2022) and miR2Disease (2877 entries, downloaded in July 2022). All human mature miRNA names were mapped to the standard miRNA accession numbers by miRBase v22.0. The disease names in the relations were mapped to the canonical disease terms in MeSH (downloaded in July 2022). We integrated the miRNA–disease interactions from miRCancer and miR2Disease and eliminated the duplicate entries. Eventually, 6099 miRNA–disease relations were obtained, involving 163 human diseases.

**Figure 2:**
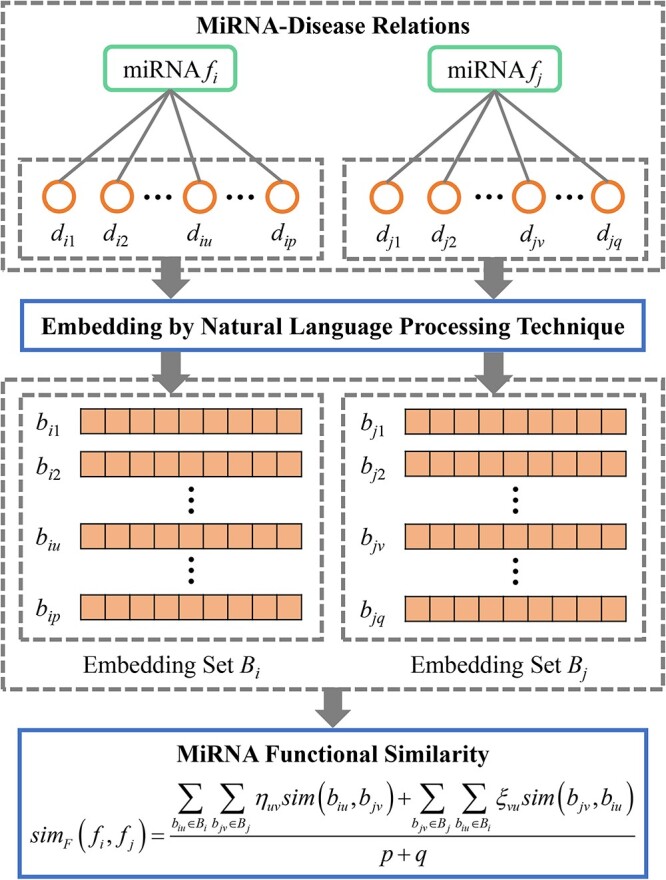
MiRNA functional similarity calculation.

### MiRNA network construction

miRMarker constructs the miRNA interaction network by integrating the cooperative regulation network ${G}_{CR}$ and the functional similarity network ${G}_{FS}$. ${G}_{CR}$ is constructed using the miRNA expression data. ${G}_{FS}$ is built based on the miRNA–disease relations.

Let $F=\left\{{f}_1,{f}_2,...,{f}_m\right\}$ represent the feature (miRNA) set and $m$ be the number of features. miRMarker defines the combination feature ${f}_{ij}={f}_i-{f}_j$ for two miRNAs ${f}_i$ and ${f}_j$ (1 ≤ *i* < *j* ≤ *m*), representing the relative expression relationship between ${f}_i$ and ${f}_j$ [31]. The dysregulation of expression level of ${f}_{ij}$ from normal samples to disease samples describes the change of cooperative regulation of ${f}_i$ and ${f}_j$. The Mann–Whitney U test was employed to measure the differential significance of ${f}_{ij}$ between the two sample groups (Kruskal–Wallis Test for multi-class samples). Then, the weight of the edge between ${f}_i$ and ${f}_j$ is defined as the negative logarithm value of the *P*-value of ${f}_{ij}\ (P-\text{value}_{ij})$ in U test. If the difference of ${f}_{ij}$ is significant between the two different sample groups, the edge weight between ${f}_i$ and ${f}_j$ is large. This means the cooperative regulation relationship between ${f}_i$ and ${f}_j$ is strong in ${G}_{CR}$.

Definition 1. Cooperative regulation network. Let ${G}_{CR}=\left\{{V}_{CR},{E}_{CR},{W}_{CR}\right\}$ be a cooperative regulation network. ${V}_{CR}=F$ is the vertex set, ${E}_{CR}=\left\{\left({f}_i,{f}_j\right)\left|{f}_i,{f}_j\in F,1\le i<j\le m\right.\right\}$ is the edge set and ${W}_{CR}=\left\{w\left({f}_i,{f}_j\right)=-\log \left(p\text{-} valu{e}_{ij}\right)\left|\left({f}_i,{f}_j\right)\in{E}_{CR}\right.\right\}$ indicates the set of edge weights.

miRMarker constructs the functional similarity network ${G}_{FS}$ based on miRNA–disease relations. Figure 2 shows the calculation process of miRNA functional similarity between ${f}_i$ and ${f}_j$. A miRNA corresponds to a regulated disease set containing many human diseases. The miRNA functional similarity between ${f}_i$ and ${f}_j$ is defined as the similarity between the corresponding regulated disease sets ${D}_i=\left\{{d}_{i1},{d}_{i2},...,{d}_{ip}\right\}$ and ${D}_j=\left\{{d}_{j1},{d}_{j2},...,{d}_{jq}\right\}$. ${d}_{iu}$(1 ≤ *u* ≤ *p*) is the u-th disease regulated by ${f}_i$. ${d}_{jv}$ (1 ≤ *v* ≤ *q*) is the *v*-th disease regulated by ${f}_j$. $p$ and $q$ are the numbers of diseases in ${D}_i$ and ${D}_j$, respectively. We employed the natural language processing (NLP) model BioBERT [32] to embed the disease terms into numeric vectors. BioBERT is a powerful NLP model trained on the massive biological and medical corpus. It can generate the high-quality embedding vectors for biomedical text. The disease embedding vectors coded the discriminative semantic information of diseases. By embedding the diseases in ${D}_i$ and ${D}_j$, we obtained the embedding sets ${B}_i=\left\{{b}_{i1},{b}_{i2},...,{b}_{ip}\right\}$ and ${B}_j=\left\{{b}_{j1},{b}_{j2},...,{b}_{jq}\right\}$. ${b}_{iu}$(1 ≤ *u* ≤ *p*) is the embedding vector of ${d}_{iu}$. ${b}_{jv}$ (1 ≤ *v* ≤ *q*) is the embedding vector of ${d}_{jv}$. If ${d}_{iu}$ and ${d}_{jv}$ are similar, the similarity between the two corresponding embedding vectors ${b}_{iu}$ and ${b}_{jv}$ is high. The cosine similarity values of all disease pairs between ${D}_i$ and ${D}_j$ constitute a $p\times q$ disease semantic similarity matrix ${M}_D$. The element in the *u*-th row and *v*-th column of ${M}_D$ indicates the cosine similarity value between ${b}_{iu}$ and ${b}_{jv}$. A weighted best matching average strategy was conducted to aggregate the disease similarity values by weighted averaging the similarity values in each row and column of ${M}_D$. The miRNA functional similarity between ${f}_i$ and ${f}_j$ is defined as follows:

(1) \begin{align*} si{m}_F\left({f}_i,{f}_j\right)&=\frac{\sum \limits_{b_{iu}\in{B}_i}\sum \limits_{b_{jv}\in{B}_j}{\eta}_{uv} sim\left({b}_{iu},{b}_{jv}\right)+\sum \limits_{b_{jv}\in{B}_j}\sum \limits_{b_{iu}\in{B}_i}{\xi}_{vu} sim\left({b}_{jv},{b}_{iu}\right)}{p+q}\\{\eta}_{uv}&=\frac{\mathit{\exp}\left( abs\left( sim\left({b}_{iu},{b}_{jv}\right)\right)\right)}{\sum \limits_{b_{jr}\in{B}_j}\mathit{\exp}\left( abs\left( sim\left({b}_{iu},{b}_{jr}\right)\right)\right)},\quad\sum \limits_{v=1}^q{\eta}_{uv}=1\nonumber\\{\xi}_{vu}&=\frac{\mathit{\exp}\left( abs\left( sim\left({b}_{jv},{b}_{iu}\right)\right)\right)}{\sum \limits_{b_{iz}\in{B}_i}\mathit{\exp}\left( abs\left( sim\left({b}_{jv},{b}_{iz}\right)\right)\right)},\quad\sum \limits_{u=1}^p{\xi}_{vu}=1\nonumber\end{align*}

where $sim\left({b}_{iu},{b}_{jv}\right)$ is the cosine similarity value between ${b}_{iu}$ and ${b}_{jv}$. ${\eta}_{uv}$ and ${\xi}_{vu}$ are the row-normalized and column-normalized weighting factors, respectively. A large $sim\left({b}_{iu},{b}_{jv}\right)$ leads to a large weighting factor ${\eta}_{uv}$. $abs\left(\right)$ indicates the absolute value function. The calculation of miRNA functional similarity $si{m}_F\left({f}_i,{f}_j\right)$ considers the similarity between each pair of the diseases in ${D}_i$ and ${D}_j$. The functional similarity between ${f}_i$ and ${f}_j$ is high if the similarity between the disease sets ${D}_i$ and ${D}_j$ is high.

Definition 2. Functional similarity network. Let ${G}_{FS}=\left\{{V}_{FS},{E}_{FS},{W}_{FS}\right\}$ be a functional similarity network. ${V}_{FS}=F$ is the vertex set, ${E}_{FS}=\left\{\left({f}_i,{f}_j\right)\left|{f}_i,{f}_j\in F,1\le i<j\le m\right.\right\}$ is the edge set of ${G}_{FS}$ and ${W}_{FS}=\left\{w\left({f}_i,{f}_j\right)= si{m}_F\left({f}_i,{f}_j\right)\left|\left({f}_i,{f}_j\right)\in{E}_{FS}\right.\right\}$ indicates the set of edge weights.

miRMarker defines the miRNA interaction network ${G}_{FN}$ by integrating the cooperative regulation network ${G}_{CR}$ and functional similarity network ${G}_{FS}$. ${G}_{CR}$ and ${G}_{FS}$ are both undirected complete graphs. All the edge weights in ${G}_{CR}$ and ${G}_{FS}$ are normalized using min–max normalization, respectively. In ${G}_{FN}$, the edge weight of $\left({f}_i,{f}_j\right)$ is the average value of normalized edge weights of $\left({f}_i,{f}_j\right)$ in ${G}_{CR}$ and ${G}_{FS}$:

(2) \begin{equation*} {w}_{FN}\left({f}_i,{f}_j\right)=\left({w}_{CR}^{\prime}\left({f}_i,{f}_j\right)+{w}_{FS}^{\prime}\left({f}_i,{f}_j\right)\right)/2 \end{equation*}

where ${w}_{CR}^{\prime}\left({f}_i,{f}_j\right)$ and ${w}_{FS}^{\prime}\left({f}_i,{f}_j\right)$ are the normalized edge weights of $\left({f}_i,{f}_j\right)$ in ${G}_{CR}$ and ${G}_{FS}$, respectively. ${G}_{FN}$ is a complete graph containing many uninformative edges with low weights. miRMarker defines a subnetwork of ${G}_{FN}$ by preserving the informative edges. The maximum spanning tree (MST) algorithm [33] is firstly performed to ensure the network connectivity. The MST of ${G}_{FN}$, ${G}_{FN- MST}$, only contains the $m-1$ edges maximizing the sum of edge weights. miRMarker links each node in ${G}_{FN- MST}$ to its *k* nearest neighbors (largest edge weights) to obtain the final miRNA interaction network ${G}_{FN- MST- kNN}$. It is connected and rich in miRNA interaction information with high weight edges.

### MiRNA module identification

miRMarker identifies the potential miRNA module biomarkers from ${G}_{FN- MST- kNN}$ based on the value-based reinforcement learning (RL) method Q-Learning [29]. It finds the optimal action selection policy by maintaining the Q-table for an agent. The Q-table records the maximum long-term rewards (*Q* values) of taking different actions at each state. Q-Learning updates the *Q* values during exploring and interacting with environment, aiming to maximize the future reward by taking a series of optimal actions based on the Q-table.

miRMarker selects the $g$ nodes with the highest comprehensive importance scores in ${G}_{FN- MST- kNN}$ as the initial state nodes. The comprehensive importance of ${f}_i$ (1 ≤ *i* ≤ *m*) is measured by combining the individual distinguishing ability and network topological importance as follows:


(3) \begin{equation*} I\left({f}_i\right)=\left({I}_e\left({f}_i\right)+{I}_t\left({f}_i\right)\right)/2 \end{equation*}

where ${I}_e\left({f}_i\right)=-\log \left(p\text{-}valu{e}_i\right)$ is the normalized expression difference score of ${f}_i$, representing the individual distinguishing ability in disease sample discrimination. $p\text{-}valu{e}_i$ is the *P*-value of ${f}_i$ in the Mann–Whitney U Test (Kruskal–Wallis Test for multi-class samples). If the expression values of ${f}_i$ show the significant difference between different sample groups, it means ${f}_i$ has a high individual distinguishing ability. ${I}_t\left({f}_i\right)= ClosenessCentrality\left({f}_i\right)$ is the normalized network topological importance score of ${f}_i$, defined as the closeness centrality of ${f}_i$ in ${G}_{FN- MST- kNN}$. Closeness centrality measures the distances from one node to all other nodes in the network. If the closeness centrality of ${f}_i$ is large, it means that ${f}_i$ is in the central position of ${G}_{FN- MST- kNN}$, the network topological importance score of ${f}_i$ is high. Both ${I}_e\left({f}_i\right)$ and ${I}_t\left({f}_i\right)$ are normalized using min–max normalization. The comprehensive importance scores of nodes consider both the individual distinguishing ability and network topological property, evaluating the node importance synthetically. The most important $g$ nodes are selected as the initial state nodes.

For an initial state node ${f}_t$ (1 ≤ *t* ≤ *m*), miRMarker defines a Q-table ${Q}_t$ with all *Q* values of zero and a set of visited nodes ${S}_t$ only containing ${f}_t$. The rows and columns of ${Q}_t$ represent the state nodes and action nodes, respectively. ${Q}_t\left({f}_s,{f}_a\right)$ indicates the *Q* value (accumulated reward) of selecting the action node ${f}_a$ when the state node is ${f}_s$. A larger *Q* value means more accumulated rewards. miRMarker selects an action node from the action space, that is, the set of neighbor nodes of the visited nodes in ${S}_t$. The epsilon–greedy strategy is adopted to handle the exploration–exploit problem. When the current state node is ${f}_s$, the action node is randomly selected from the action space with a probability $\varepsilon$. Otherwise, miRMarker selects the action node ${f}_a$ leading to the largest ${Q}_t\left({f}_s,{f}_a\right)$. $\varepsilon$ is decayed with episodes as follows:


(4) \begin{equation*} \varepsilon ={\varepsilon}_f^{e/K} \end{equation*}

where ${\varepsilon}_f$ is a small value, typically set as 1e−05 [28]. $e$ is the current episode number. $K$ indicates the maximum number of episodes. miRMarker fully explores the action space in the early episodes, tending to select a random action node. $\varepsilon$ decreases exponentially as $e$ increases. miRMarker gradually reduces the selection of random action nodes but selects the optimal action node, that is, the node leading to the max *Q* value in action space. The selected action node ${f}_a$ is added to ${S}_t$.

The reward value of adding ${f}_a$ to ${S}_t$ considers the classification performance and size of ${S}_t$ simultaneously as follows:


(5) \begin{equation*} R\left({S}_t\right)= AUC\left({S}_t\right)\times SF\left({S}_t\right) \end{equation*}

where $AUC\left({S}_t\right)$ is the AUC value of ${S}_t$ in ROC analysis. $SF\left({S}_t\right)=1+ sqrt\left(1/\left|{S}_t\right|\right)$ indicates the size factor of ${S}_t$. $sqrt\left(\right)$ is the square root function. $\left|{S}_t\right|$ indicates the size of set ${S}_t$. The reward value $R\left({S}_t\right)$ is large if ${S}_t$ has a high classification performance and a small size. ${Q}_t\left({f}_s,{f}_a\right)$ is updated as follows:


(6) \begin{equation*} {Q}_t\left({f}_s,{f}_a\right)={Q}_t\left({f}_s,{f}_a\right)+\alpha \left(R\left({S}_t\right)+\gamma \max \left({Q}_t\left({f}_a,{f}_a^{\prime}\right)\right)-{Q}_t\left({f}_s,{f}_a\right)\right) \end{equation*}

where $\alpha$ is the learning rate and $\gamma$ is the discount rate. ${Q}_t\left({f}_s,{f}_a\right)$ is updated greatly if $\alpha$ is large, meaning the information learned by interacting with the environment play a major role. If $\alpha$ is small, ${Q}_t\left({f}_s,{f}_a\right)$ gets a few updates. $\max \left({Q}_t\left({f}_a,{f}_a^{\prime}\right)\right)$ indicates the maximum *Q* value of selecting any action node ${f}_a^{\prime }$ when the state node is ${f}_a$. The updating of ${Q}_t\left({f}_s,{f}_a\right)$ makes use of the immediate reward, the discounted maximum expected accumulated reward and the current estimation of the *Q* value simultaneously. ${f}_s$ is updated using the latest action node ${f}_a$. Then, the new action node is selected iteratively. The current episode stops when $AUC\left({S}_t\right)$ reaches the maximum value of 1.000 or $\left|{S}_t\right|$ exceeds a predefined limit. At the end of all the episodes, miRMarker has explored the miRNA network sufficiently and learned the valuable information for optimal action policy.

miRMarker defines the essential miRNA modules using the learned Q-table. The module extension process starts from each initial state node ${f}_t$. Initially, the module ${M}_t$ only contains ${f}_t$. The optimal action node ${f}_a$ was selected iteratively using the epsilon–greedy strategy based on the learned Q-table. The selected action node ${f}_a$ is added to ${M}_t$. The state node is changed to ${f}_a$. There are two conditions of stopping the module extension: (i) the AUC of module ${M}_t$ reaches the maximum value of 1.000, which means the performance of ${M}_t$ has no further improvement, and (ii) the new action node ${f}_a$ has been added to ${M}_t$ during the module extension. No new node will be added in this situation.

Algorithm 1 shows the pseudocode of module identification in miRMarker. Lines 1–2 initialize the visited node set ${S}_t$ and Q-table ${Q}_t$, respectively. Line 3 initializes an episode. Line 4 sets the $\varepsilon$ at the current episode. Line 5 starts a search procedure. Line 6 defines the state node. Line 7 selects the action node by the epsilon–greedy strategy. Line 8 adds the action node to ${S}_t$. Line 9 evaluates the classification performance of ${S}_t$. Line 10 calculates the reward value. Line 11 updates the Q-table. Lines 12–14 judge the stop conditions of searching. Line 17 identifies the module based on the learned Q-table.

**Algorithm 1:** Module Identification in miRMarker.

**Input:** Graph *G* = (*V*, *E*), Training set *X*, Number of episodes *K*, Initial state node *f_t_*, Learning rate *α*, Size limit *L*.	
**Output:** Defined module *M_t_*.	
1	Initialize the visited node set *S_t_* = {*f_t_*};
2	Initialize the Q-table *Q_t_*;
3	**for** *e* = 1, 2, …, *K* **do**:
4	*ε* = *DecayProbability*(*K*, *e*);
5	**while(true):**
6	*f_s_* = the last node added to *S_t_*;
7	*f_a_* = *EpsilonGreedyStrategy*(*M_t_*, *G*, *Q_t_*, *ε*);
8	*S_t_* = *S_t_* ∪ {*f_a_*};
9	*AUC*(*S_t_*) = *ModulePerformance*(*X*, *S_t_*);
10	*R*(*S_t_*) = *RewardEvaluation*(*S_t_*, *AUC*(*S_t_*));
11	*Q_t_*(*f_s_*, *f_a_*) = *Q_t_*(*f_s_*, *f_a_*) + *α* ^*^ (*R*(*S_t_*) + *γ* ^*^ max(*Q_t_*(*f_a_*, *f_a_*’)) − *Q_t_*(*f_s_*, *f_a_*));
12	**if** *AUC*(*S_t_*) = 1 or |*S_t_*| ≥ *L* **do**:
13	** break**;
14	** end**
15	** end**
16	**end**
17	*M_t_* = *ModuleExtensionByQTable*(*G*, *X*, *Q_t_*, *f_t_*);
18	**return** *M_t_*.

miRMarker identifies one module for each of the $g$ initial state nodes. On each identified module, a support vector machine (SVM) classifier [34] with a linear kernel is built. The weighted voting is employed to integrate the prediction probability values of $g$ classifiers. The weights of $g$ classifiers are defined using the normalized average AUCs of modules in an inner 5-fold cross-validation on training samples. Then, miRMarker labels new samples by the weighted voting of the $g$ classifiers.

### Involved statistics

Several statistics are used in the proposed method. In constructing the cooperative regulation network, the Mann–Whitney U test is employed to measure the differential significance of the combinatorial feature ${f}_{ij}$ between the two different sample groups (Kruskal–Wallis Test for multi-class samples). In the construction of a functional similarity network, the cosine similarity is adopted to calculate the disease semantic similarity between two disease embedding vectors. In module identification, the Mann–Whitney U test is adopted to calculate the individual distinguishing ability of features for defining the initial nodes (Kruskal–Wallis test for multi-class samples). Large differences of feature expression levels between the two groups indicate the high individual distinguishing ability of a feature.

### Experimental settings

We compared miRMarker with the efficient multivariable feature selection method SVM-recursive feature elimination (SVM-RFE) [35] and the network-based data analysis methods WGCNA, DNB, GroupBN [36], NGTM, QLCD and defining disease-related modules (DDRM) [19] to show its validation.

SVM-RFE is an efficient feature selection method that integrates the SVM and recursive feature elimination to select the informative features. DNB was proposed for time series data analysis [11]. In this study, we adopted DNB to analyze the static data. The hierarchical clustering was employed to obtain the potential feature groups firstly [11]. Then, DNB constructed the Pearson correlation networks on each sample group, respectively, and calculated the critical index (CI) score for each feature group by evaluating the changes of molecule correlation and the SD between different correlation networks. The feature group with the maximum CI score is defined as the DNB biomarkers. GroupBN employs the hierarchical clustering technique to group the similar features and constructs the Bayesian network to model the relationships between the feature groups for probabilistic inference. DDRM evaluates the miRNA synergistic relationships by considering the co-regulated target genes and non-co-regulated targets simultaneously and then constructs the weighted miRNA synergistic network. It found 200 miRNA functional modules using the kernel clustering method. For a specific disease study, DDRM maps the corresponding miRNA expression data to the miRNA functional modules, evaluates each module based on the mapped miRNAs and defines the key modules for the disease. We adopted the top five functional modules with the maximum average performance in an inner 5-fold cross-validation on training samples as the DDRM biomarkers. NGTM was proposed for known gene regulatory networks [26]. QLCD was designed for existing complex network datasets [28]. For NGTM and QLCD, we constructed the correlation networks based on the miRNA datasets by symmetrical uncertainty (SU) [37]. SU is a widely used entropy-based similarity measure. Each node was linked to its $k$ neighbors with the highest SU values. The $k$ value was searched in {1, 3, 5, 7, 9} to make the node degrees in SU network fit the power law distribution best.

In this study, the NLP technique was adopted to obtain the disease semantic vectors for calculating the miRNA functional similarity based on the known miRNA–disease relations. Thus, we also compared miRMarker with the method using the disease DAG-based miRNA functional similarity network (named as N-DAG) [23], aiming to show the effectiveness of miRNA functional similarity calculation in miRMarker.

The effectiveness of miRMarker was evaluated on binary classification and multi-class classification, respectively, for distinguishing the different sample groups. The receiver operating characteristic (ROC) analysis was conducted to evaluate the classification performance (macro-averaged one-versus-rest ROC for multi-class classification). The 10-fold cross-validation was performed 10 times to get the average area under the curve (AUC), sensitivity and specificity.

Dataset GSE32273 consists of the samples from three different tissues, thus, it was split into three subsets depending on tissue types. For binary classification, we also used the multi-class datasets (GSE31164, GSE39046 and GSE35834) by dividing them into multiple binary subsets. Consequently, there were a total of 17 binary datasets/subsets included in the binary classification.

In miRMarker, the $k$ value was searched in {3, 5, 7, 9} to make the node degrees in ${G}_{FN- MST- kNN}$ fit the power law distribution best. The maximum episode number $K$ in module identification was set as 2000. The learning rate $\alpha$ was set as 0.2. The discount rate $\gamma$ was set as 0.8. The number of initial state nodes was set as 5. The size limit of the visited node set ${S}_t$ is set as 50.

For all the feature selection methods, the SVM with linear kernel was adopted as the classifier. Unit-variance (UV) scaling was performed to nondimensionalize the features before training the models. All methods were implemented in R 4.2.0.

## RESULTS AND DISCUSSION

### Performance evaluation of miRMarker

We compared miRMarker with eight efficient data analysis methods SVM-RFE, WGCNA, DNB, GroupBN, NGTM, QLCD, N-DAG and DDRM in ROC analysis. Table 2 shows the comparison results in AUC in binary classification. Bold font marks the highest AUC values on the corresponding dataset. Mark ‘^*^’ means the AUC value of the method is significantly different from that of miRMarker (*t*-test, *P-*value < 0.05). The row ‘Average’ shows the average AUC values for each method over the 17 binary datasets. The row ‘Win/Tie/Loss’ lists the numbers of datasets on which the average performance of miRMarker is significantly higher than, not significantly different from, or significantly less than that of the comparison method.

**Table 2:** Comparison on the miRNA datasets in AUC

Datasets	miRMarker	SVM-RFE	WGCNA	DNB	GroupBN	NGTM	QLCD	N-DAG	DDRM
GSE31164–1	0.743 ± 0.161	0.721 ± 0.157	0.690 ± 0.143^*^	0.683 ± 0.141^*^	**0.804 ± 0.135** ^*^	0.671 ± 0.141^*^	0.694 ± 0.145^*^	0.738 ± 0.152	0.724 ± 0.135
GSE31164–2	0.888 ± 0.168	0.857 ± 0.185	**0.893 ± 0.163**	0.743 ± 0.175^*^	0.686 ± 0.125^*^	0.738 ± 0.184^*^	0.773 ± 0.198^*^	0.873 ± 0.179	0.855 ± 0.190
GSE31164–3	**0.767 ± 0.170**	0.705 ± 0.168^*^	0.698 ± 0.159^*^	0.691 ± 0.142^*^	0.587 ± 0.044^*^	0.714 ± 0.159^*^	0.673 ± 0.136^*^	0.704 ± 0.149^*^	0.699 ± 0.144^*^
GSE39046–1	**0.962 ± 0.086**	0.922 ± 0.134^*^	0.916 ± 0.145^*^	0.717 ± 0.167^*^	0.910 ± 0.145^*^	0.838 ± 0.168^*^	0.934 ± 0.120^*^	0.953 ± 0.117	0.928 ± 0.145^*^
GSE39046–2	**0.983 ± 0.064**	0.924 ± 0.127^*^	0.961 ± 0.092	0.852 ± 0.184^*^	0.733 ± 0.204^*^	0.828 ± 0.186^*^	0.843 ± 0.160^*^	0.981 ± 0.063	0.978 ± 0.065
GSE39046–3	0.971 ± 0.081	0.941 ± 0.127	**0.972 ± 0.079**	0.864 ± 0.183^*^	0.884 ± 0.162^*^	0.832 ± 0.179^*^	0.807 ± 0.202^*^	0.923 ± 0.140^*^	0.933 ± 0.131^*^
GSE41574	0.968 ± 0.088	0.930 ± 0.143^*^	0.934 ± 0.150^*^	0.798 ± 0.182^*^	0.785 ± 0.221^*^	0.808 ± 0.193^*^	0.919 ± 0.150^*^	0.961 ± 0.102	**0.988 ± 0.054** ^*^
GSE67139	0.933 ± 0.071	0.850 ± 0.119^*^	0.896 ± 0.098^*^	0.785 ± 0.159^*^	0.874 ± 0.120^*^	0.846 ± 0.133^*^	0.892 ± 0.094^*^	**0.934 ± 0.082**	0.864 ± 0.124^*^
GSE32273–1	**0.939 ± 0.125**	0.917 ± 0.153	0.928 ± 0.140	0.918 ± 0.164	0.925 ± 0.162	0.804 ± 0.203^*^	0.917 ± 0.160	0.913 ± 0.154	0.912 ± 0.158^*^
GSE32273–2	0.801 ± 0.187	0.746 ± 0.198^*^	**0.893 ± 0.177** ^*^	0.785 ± 0.195	0.828 ± 0.200	0.774 ± 0.198	0.735 ± 0.203^*^	0.824 ± 0.188	0.755 ± 0.210
GSE32273–3	**0.830 ± 0.198**	0.808 ± 0.183	0.775 ± 0.199^*^	0.805 ± 0.195	0.744 ± 0.151^*^	0.745 ± 0.201^*^	0.782 ± 0.206	0.810 ± 0.187	0.756 ± 0.193^*^
GSE34496	0.968 ± 0.077	0.931 ± 0.124^*^	0.922 ± 0.105^*^	0.783 ± 0.176^*^	**0.981 ± 0.053** ^*^	0.871 ± 0.147^*^	0.920 ± 0.128^*^	0.946 ± 0.092^*^	0.976 ± 0.065
GSE35834–1	**0.993 ± 0.032**	0.984 ± 0.060	**0.993 ± 0.030**	0.827 ± 0.182^*^	0.984 ± 0.058	0.868 ± 0.160^*^	0.962 ± 0.104^*^	0.958 ± 0.105^*^	0.983 ± 0.059
GSE35834–2	**1.000 ± 0.000**	0.970 ± 0.094^*^	0.986 ± 0.054^*^	0.781 ± 0.209^*^	0.970 ± 0.107^*^	0.902 ± 0.171^*^	0.986 ± 0.054^*^	0.949 ± 0.115^*^	0.993 ± 0.039
GSE35834–3	**0.878 ± 0.163**	0.816 ± 0.186^*^	0.744 ± 0.175^*^	0.734 ± 0.179^*^	0.768 ± 0.150^*^	0.749 ± 0.168^*^	0.838 ± 0.184	0.768 ± 0.191^*^	0.803 ± 0.185^*^
GSE41282	0.928 ± 0.148	**0.955 ± 0.120**	0.880 ± 0.190^*^	0.740 ± 0.207^*^	0.869 ± 0.197^*^	0.790 ± 0.206^*^	0.895 ± 0.189	0.848 ± 0.210^*^	0.855 ± 0.202^*^
GSE108153	**0.985 ± 0.060**	0.933 ± 0.149^*^	0.984 ± 0.073	0.913 ± 0.155^*^	0.913 ± 0.156^*^	0.849 ± 0.189^*^	0.943 ± 0.132^*^	0.976 ± 0.091	0.970 ± 0.103
Average	**0.914 ± 0.110**	0.877 ± 0.143	0.886 ± 0.128	0.789 ± 0.176	0.838 ± 0.141	0.802 ± 0.176	0.854 ± 0.151	0.886 ± 0.136	0.881 ± 0.130
Win/Tie/Loss	#	10/7/0	10/6/1	14/3/0	12/3/2	16/1/0	13/4/0	7/10/0	8/8/1

miRMarker performed better than other comparison methods on most cases. Compared with SVM-RFE, miRMarker outperformed it on 10 of the 17 datasets in AUC. In comparison with the network-based data analysis methods, miRMarker defeated WGCNA, DNB, GroupBN, NGTM, QLCD and DDRM on 10, 14, 12, 16, 13 and 8 datasets, respectively. miRMarker outperformed N-DAG on 7 datasets and tied with it on 10 datasets. It can be seen that miRMarker achieved the highest AUCs (bold fonts) on 9 of the 17 datasets. Besides, miRMarker obtained the highest average AUC (0.914) over the different datasets and the lowest average SD (0.110). Tables S1 and S2, available online at http://bib.oxfordjournals.org/, show the comparison results in sensitivity and specificity, respectively. It can be seen that miRMarker obtained the highest values on eight and eight datasets, respectively. In addition, miRMarker obtained the highest average sensitivity (0.948) and specificity (0.922) over datasets. Additionally, we examined the comparison results between miRMarker and other data analysis methods in the Matthews correlation coefficient (MCC) (Table S3 available online at http://bib.oxfordjournals.org/). miRMarker got the highest average MCC (0.662) over datasets. The comparison results in binary classification illustrated the effectiveness of miRMarker. Table S4 available online at http://bib.oxfordjournals.org/ gives the comparison results for multi-class classification. The comparison results in multi-class classification also indicated that miRMarker showed more advantageous performance than other methods.

miRMarker integrates the multi-view miRNA networks constructed on the expression data and the known miRNA–disease relations, achieving the robust miRNA fusion network. The important miRNA modules are defined by the reinforcement learning strategy. In a word, miRMarker can make full use of the miRNA interaction information hidden in the expression data and miRNA–disease relations, as well as define the essential miRNA module biomarkers for disease diagnosis and prognosis.

**Figure 3:**
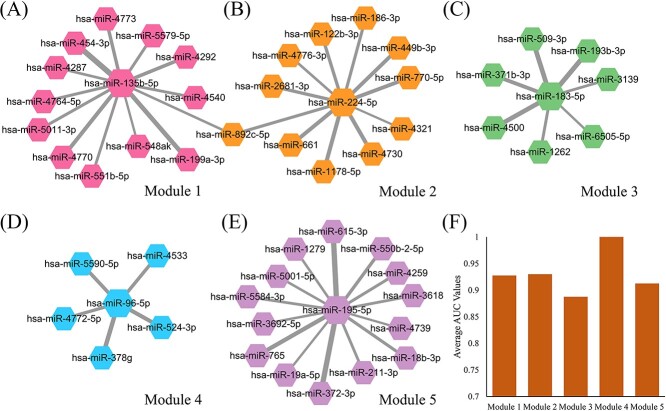
Identified modules on colorectal cancer dataset and their performance in disease sample discrimination. (**A**–**E**) Identified modules (Modules 1–5). The thickness of the edge indicates the edge weights. (**F**) Average AUC values of Modules 1–5 in the 5-fold cross validation on training samples.

### Ablation study

miRMarker builds the miRNA interaction network by integrating the cooperative regulation network and the functional similarity network. We compared miRMarker with the methods only use the cooperative regulation network (named as N-CR) or functional similarity network (named as N-FS) for validating the effectiveness of network integration. Figure S1 available online at http://bib.oxfordjournals.org/ shows the comparison results in average AUC values over datasets. miRMarker obtained better performance than N-CD and N-FS. The integration of the cooperative regulation network and functional similarity network uses the multi-view miRNA interactions well to obtain the robust miRNA interactions. It is helpful for finding the critical information for disease sample discrimination.

**Figure 4:**
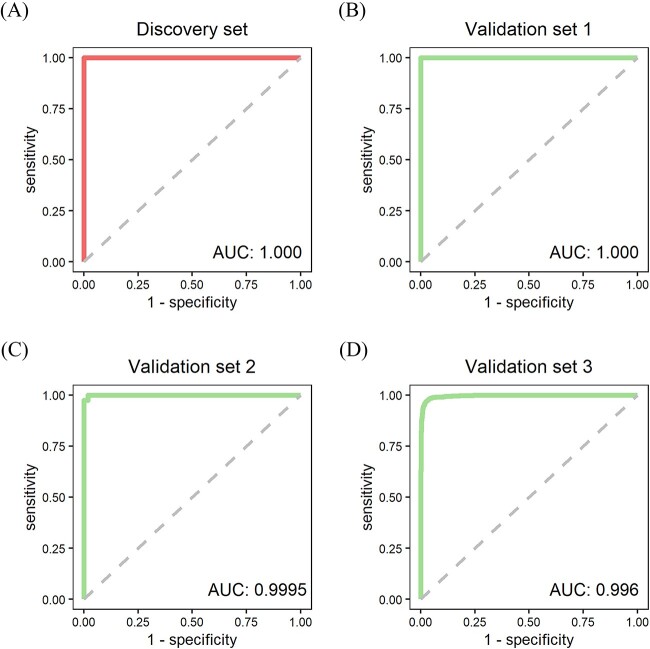
ROC curves of miRMarker on colorectal cancer datasets. (**A**) ROC cure on the discovery set (GSE108153). (**B**–**D**) ROC cures on the independent validations sets from GSE112264, GSE113486 and GSE211692, respectively.

### Parameter sensitivity analysis

There are two main parameters in miRMarker: (i) the learning rate $\alpha$ and (ii) the number of initial state nodes $g$ (module numbers). The setting of $\alpha$ concerns the update range of *Q* values in the module identification. Figure S2A available online at http://bib.oxfordjournals.org/ shows the average AUC values of miRMarker for different settings of $\alpha$ in {0.2, 0.4, 0.6, 0.8}. It can be seen that the performance of miRMarker decreases slightly when $\alpha$ changes from 0.2 to 0.8, meaning miRMarker is relatively stable under the different settings of $\alpha$. A small learning rate $\alpha$ may result in better performance, which is consistent with the previous study [28]. $\alpha =0.2$ was adopted in this study.

The setting of module number $g$ influences the effects of ensemble strategy in disease sample discrimination. Figure S2B available online at http://bib.oxfordjournals.org/ shows the average AUC values of miRMarker for different settings of $g$ in {1, 3, 5, 7}. The performance of miRMarker shows great improvements continuously as $g$ changes from 1 to 5. However, the performance change of miRMarker becomes flat when $g$ changes from 5 to 7, indicating the performance is hard to improve further. $g=5$ is applicable to most cases and adopted in this study.

### Biomarker identification on colorectal cancer dataset

We performed miRMarker to identify the potential miRNA biomarkers for colorectal cancer diagnosis on a real-world transcriptomics dataset from the GEO database (GSE108153). Colorectal cancer is a global malignant tumor facing many risk factors such as smoking, unhealthy diet and obesity [38]. Current treatment methods for colorectal cancer, including radiotherapy and resection, have limited influences on the cure rate and prognosis. The improvement in diagnosis and prognosis of colorectal cancer is still challenging and attractive.

The discovery set (GSE108153) contains 21 pairs of cancer and adjacent normal tissues collected from the colorectal cancer patients. Three cancer transcriptomics datasets (GSE112264, GSE113486, GSE211692) involving multiple solid cancers were retrieved from the GEO database. For each of them, we extracted the colorectal cancer samples and non-cancer samples as the independent validation sets. Table S5 available online at http://bib.oxfordjournals.org/ gives the details of the three independent validation sets. The identified potential biomarkers by miRMarker on the discovery set were validated on the independent validation sets.

miRMarker defined five miRNA modules on the discovery set (Figure 3A–E). Each of the identified modules is a star graph. They showed good performance in distinguishing the cancer samples from normal samples (Figure 3F).

Figure 4 shows the ROC curves of miRMarker on the discovery set and three independent validation sets. For distinguishing the colorectal cancer samples from normal samples, miRMarker obtained an AUC of 1.000 on the discovery set. On the three independent validation sets, the AUC values were 1.000, 0.9995 and 0.996, respectively. The defined modules performed outstandingly in cancer sample discrimination on the discovery set and independent validation sets, showing the great potential as the biomarkers for colorectal cancer.

**Figure 5:**
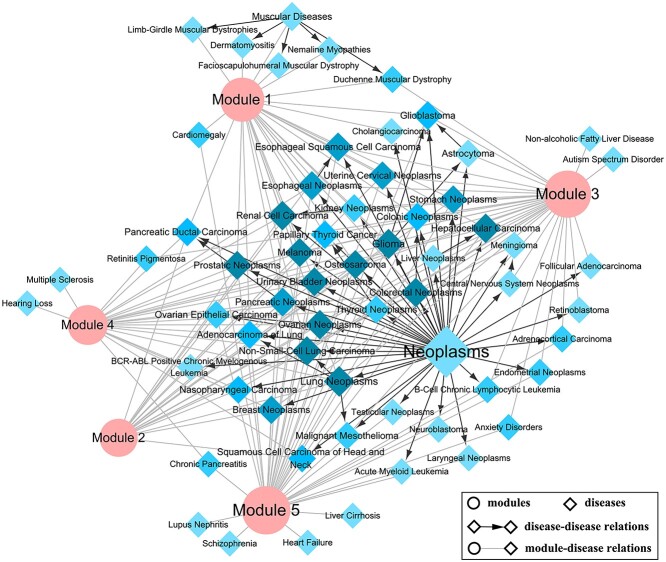
Module–disease network. Node sizes represent the node degree. Arrow direction in disease–disease edges indicates that the source node includes the target node.

Furthermore, we explored the relationships between the defined miRNA modules and colorectal cancer by constructing the module–disease network (Figure 5). All the five defined modules are connected to the disease node of colorectal neoplasms, reflecting that the defined modules are closely related to the occurrence and development of colorectal neoplasms. More details about the module–disease network are given in Supplementary Materials available online at http://bib.oxfordjournals.org/.

**Figure 6:**
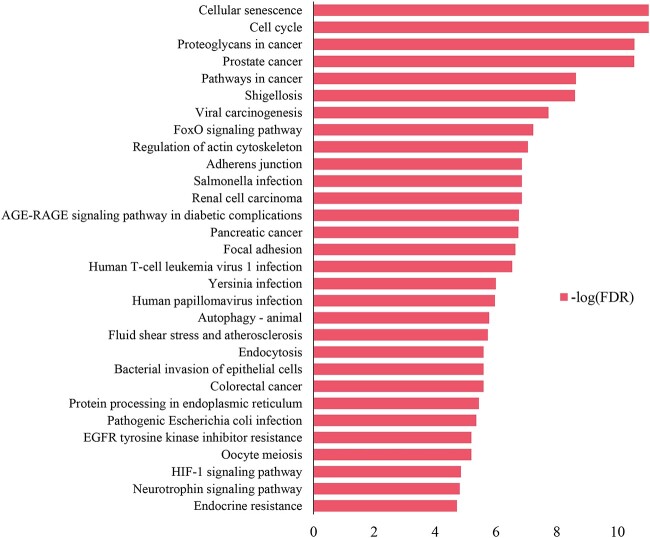
Top 30 enriched KEGG pathways of target genes of hub miRNAs.

Highly connected hub nodes usually locate at the key positions in biological networks, which play important roles in biological processes [39]. The pathway analysis was conducted for the target genes of the five hub miRNAs (hsa-miR-135b-5p, hsa-miR-224-5p, hsa-miR-183-5p, hsa-miR-96-5p and hsa-miR-195-5p) using the online tool DAVID [40, 41]. Figure 6 shows the top 30 enriched pathways of Kyoto Encyclopedia of Genes and Genomes (KEGG) [42]. Most of the top enriched pathways are associated with cellular growth and senescence, signal transduction, viral or bacterial infection and cancer-related metabolism. Typically, 51 target genes were enriched in the KEGG pathway ‘Colorectal cancer’ (hsa05210) with a false discovery rate (FDR) of 2.55e−06 (Table S6 available online at http://bib.oxfordjournals.org/), indicating the significant roles of the regulated target genes in the occurrence and development of colorectal cancer.

To further understand the mechanism of miRNA regulating genes in colorectal cancer development, we performed the gene expression analysis using The Cancer Genome Atlas (TCGA) Colon Cancer cohort. The TCGA gene expression data of colon cancer were derived from the UCSC Xena platform (http://xena.ucsc.edu/). Forty-one normal samples and 453 primary colon tumor samples were included in the gene expression analysis. Figure S3 available online at http://bib.oxfordjournals.org/ shows the overview of dysregulated pathway ‘Colorectal cancer’ (hsa05210). The dysregulation of target genes in colorectal cancer involves the important cellular function pathways ‘Cell cycle’ (hsa04110, enrichment FDR = 5.01e−12), ‘Apoptosis’ (hsa04210, enrichment FDR = 3.75e−03) and several critical signaling pathways, including the Wnt signaling pathway (hsa04310, enrichment FDR = 2.20e−04), MAPK signaling pathway (hsa04010, enrichment FDR = 3.78e−05) and p53 signaling pathway (hsa04115, enrichment FDR = 2.36e−05). It can be seen that the dysregulation of one hub miRNA causes the disturbance of multiple colorectal cancer-related pathways, promoting the development of colorectal cancer further jointly. More details about the gene expression analysis in pathways are given in Supplementary Materials available online at http://bib.oxfordjournals.org/.

Cancer development involves various biological processes. Different pathways interact with each other and influence the tumor process together. There are complex regulatory relationships between miRNAs and target genes. The experimental results showed that the identified miRNA modules not only have the outstanding ability in disease sample discrimination but also play critical roles in the occurrence and development of colorectal cancer by participating in the perturbations of multiple related pathways. The identified miRNAs are promising as potential colorectal cancer biomarkers in clinical applications. More experiments are required to validate the biological significances of the identified miRNAs in colorectal cancer.

### Experiments on other diseases

In addition, we included another three experiments for further verifying the effectiveness of miRMarker. Three challenging tasks were considered: the prognosis of nasopharyngeal carcinoma (NPC), the diagnosis of recurrent implantation failure (RIF) and the severity judgment of COVID-19. Table S7 available online at http://bib.oxfordjournals.org/ gives the details of the involved discovery sets and independent validation sets.

As performed for colorectal cancer, the identified potential biomarkers by miRMarker on the discovery set were validated on the corresponding independent validation set. Table S8 available online at http://bib.oxfordjournals.org/ shows the results of the three experiments. For the NPC experiment, miRMarker got an AUC of 0.922 on the discovery set and an AUC of 0.731 on the independent validation set. For the RIF experiment, the AUC values of miRMarker on the discovery set and independent validation set were 0.989 and 0.967, respectively. For the COVID-19 experiment, miRMarker got an AUC of 0.895 on discovery set and an AUC of 1.000 on the independent validation set.

The results of the three experiments further illustrated the effectiveness of miRMarker in identifying the potential biomarkers for different diseases. More details about the three experiments are given in Supplementary Materials available online at http://bib.oxfordjournals.org/. In summary, miRMarker demonstrates its advantages in disease biomarker identification.

## CONCLUSION

Identifying the informative miRNAs for the diagnosis and prognosis of diseases, such as cancers, is attractive and challenging. Network-based data analysis methods are important tools in potential biomarker identification. In this study, we proposed a miRNA data analysis method based on multi-view networks and reinforcement learning (miRMarker) to define the potential miRNA biomarkers for diseases. miRMarker constructs the cooperative regulation network and functional similarity networks based on the miRNA expression data and known miRNA–disease relations, respectively. Then, reinforcement learning strategy is employed to define the potential miRNA module biomarkers for discriminating different sample groups.

We compared miRMarker with eight efficient data analysis methods SVM-RFE, WGCNA, DNB, GroupBN, NGTM, QLCD, N-DAG and DDRM on multiple miRNA datasets. miRMarker obtained better performance in disease sample discrimination, indicating that it identified the valuable miRNAs based on the miRNA expression data and knowledge bases. In addition, we applied miRMarker to define the potential biomarkers for colorectal cancer. The defined miRNA modules play critical roles in the development of colorectal cancer by regulating multiple related pathways. The experimental results illustrated the identified miRNAs of miRMarker have the outstanding ability in disease sample discrimination, as well as the significant biological importance in the development of colorectal cancer. Integrating the multi-view miRNA networks is conducive to construct the robust miRNA interaction network. Besides, defining the miRNA modules by reinforcement learning can fully explore the network information and identify the more competitive miRNA biomarkers for disease sample discrimination. In conclusion, miRMarker is promising in miRNA data analysis for human diseases.

It is necessary to acknowledge the limitations of this work. The miRNA functional similarity network relies on the known miRNA–disease relations in the public knowledge bases. The further updates and enriching of public knowledge bases will benefit the evaluation of miRNA functional similarity. Meanwhile, the constructed miRNA functional similarity network is designed to be suitable for different diseases, which may fail to highlight the specific characteristics of some diseases. A possible improvement is to consider the construction of the specific functional interaction network for a certain disease. Besides, in the future work, we plan to develop the parallel version of miRMarker to further improve the efficiency of disease biomarker identification.

**Key Points**

- The miRNA interaction network is constructed by integrating the multi-view miRNA networks based on expression data and known miRNA–disease relations.
- Natural language processing is adopted in calculating the miRNA functional similarity based on the known miRNA–disease relations.
- Important miRNA modules related to disease development are defined using the reinforcement learning strategy.
- The comparison results with eight data analysis methods illustrated the effectiveness of miRMarker.
- The defined modules not only have outstanding ability in disease sample discrimination but also play critical roles in the perturbations of related pathways.

## Supplementary Material

supplementary_materials-mirmarker-20231102-clean-2_bbad427

table_s4_discussion_bbad427

## Data Availability

The transcriptomics datasets are available in Gene Expression Omnibus (GEO, https://www.ncbi.nlm.nih.gov/geo/). The TCGA genomics datasets can be accessed from UCSC Xena platform (http://xena.ucsc.edu/). The R package of miRMarker can be available at https://github.com/DLUT-datas/miRMarker.

## Author Biographies

**Benzhe Su** is a PhD candidate in School of Computer Science and Technology, Dalian University of Technology. His research interests include biological data analysis and machine learning methods in bioinformatics.

**Weiwei Wang** is a master student in School of Computer Science and Technology, Dalian University of Technology. Her research interests include bioinformatics and machine learning.

**Xiaohui Lin** is a professor in the School of Computer Science and Technology, Dalian University of Technology. Her research focus is on the algorithm design and analysis, machine learning, biological information processing.

**Shenglan Liu** is an associate professor in the School of Innovation and Entrepreneurship, Dalian University of Technology. His research focus is on perception computing and machine learning.

**Xin Huang** is an associate professor in the School of Mathematics and Information Science, Anshan Normal University. His research interests include bioinformatics and machine learning.
