# Supplementary material for: Identifying the potential miRNA biomarkers based on multi-view networks and reinforcement learning for diseases
Source: Brief Bioinform. 2023 Nov 28;25(1):bbad427. doi: 10.1093/bib/bbad427 (PMC10753537; doi:10.1093/bib/bbad427)
Supplement: supplementary_materials-mirmarker-20231102-clean-2_bbad427 [file supplementary_materials-mirmarker-20231102-clean-2_bbad427.docx]

**Identifying the potential miRNA biomarkers based on multi-view networks and reinforcement learning for diseases**

Benzhe Su^1^, Weiwei Wang^1^, Xiaohui Lin^1,*^, Shenglan Liu^2^, Xin Huang^3^

^1^ School of Computer Science and Technology, Dalian University of Technology, Dalian 116024, Liaoning, China.

^2^ School of Innovation and Entrepreneurship, Dalian University of Technology, Dalian 116024, Liaoning, China.

^3^ School of Mathematics and Information Science, Anshan Normal University, Anshan 114007, Liaoning, China.

* Address correspondence to:

Prof. Xiaohui Lin, School of Computer Science and Technology, Dalian University of Technology, Dalian 116024, Liaoning, China. E-mail: datas@dlut.edu.cn.

**MiRNA Names Mapping**

For each dataset, the human mature miRNA names were mapped to the standard miRNA accession numbers (MIMAT IDs) using the miRBase database. The miRNAs with valid miRNA names were kept. For each valid miRNA, the unique MIMAT ID was mapped. These miRNAs were eliminated if their MIMAT IDs were not used in the latest miRBase, which means that they are not considered as miRNAs anymore. The miRNA name mapping process was completed using R package “miRNAmeConverter”.

Table S1. Comparison on the miRNA datasets in sensitivity.

| Datasets | miRMarker | SVM-RFE | WGCNA | DNB | GroupBN | NGTM | QLCD | N-DAG | DDRM |
| --- | --- | --- | --- | --- | --- | --- | --- | --- | --- |
| GSE31164-1 | 0.768±0.191 | 0.753±0.183 | 0.698±0.186* | 0.725±0.189 | **0.863±0.221*** | 0.710±0.178* | 0.710±0.192* | 0.770±0.167 | 0.748±0.187 |
| GSE31164-2 | 0.925±0.179 | **0.945±0.157** | 0.940±0.163 | 0.885±0.211 | **0.945±0.157** | 0.845±0.232* | 0.885±0.211 | 0.940±0.163 | 0.925±0.179 |
| GSE31164-3 | **0.935±0.169** | 0.885±0.211 | 0.870±0.220* | 0.855±0.228* | 0.520±0.098* | 0.870±0.220* | 0.840±0.234* | 0.920±0.184 | 0.895±0.205 |
| GSE39046-1 | **0.972±0.098** | 0.940±0.135* | 0.943±0.139 | 0.848±0.174* | 0.938±0.151 | 0.903±0.168* | 0.968±0.102 | 0.958±0.122 | 0.937±0.144* |
| GSE39046-2 | 0.995±0.050 | 0.957±0.139* | 0.967±0.123* | 0.930±0.164* | 0.875±0.210* | 0.915±0.184* | 0.922±0.176* | **1.000±0.000** | 0.995±0.050 |
| GSE39046-3 | 0.983±0.084 | **1.000±0.000*** | 0.992±0.060 | 0.925±0.176* | 0.945±0.155* | 0.897±0.194* | 0.867±0.220* | 0.943±0.150* | 0.975±0.102 |
| GSE41574 | 0.973±0.091 | 0.940±0.151* | 0.945±0.142* | 0.835±0.190* | 0.927±0.154* | 0.855±0.199* | 0.950±0.126 | **0.983±0.073** | **0.983±0.073** |
| GSE67139 | **0.919±0.121** | 0.839±0.168* | 0.894±0.146 | 0.828±0.174* | 0.872±0.142* | 0.848±0.146* | 0.861±0.146* | 0.899±0.117 | 0.851±0.159* |
| GSE32273-1 | **0.968±0.110** | 0.935±0.153* | 0.960±0.126 | 0.957±0.135 | 0.948±0.138 | 0.913±0.192* | 0.945±0.155 | 0.945±0.154 | 0.942±0.149 |
| GSE32273-2 | 0.907±0.186 | 0.858±0.218 | 0.930±0.166 | 0.888±0.208 | 0.882±0.212 | 0.915±0.170 | 0.882±0.212 | **0.950±0.141*** | 0.838±0.228* |
| GSE32273-3 | **0.917±0.180** | 0.887±0.204 | 0.872±0.209 | 0.910±0.180 | 0.875±0.204 | 0.882±0.206 | 0.908±0.189 | 0.913±0.180 | 0.847±0.227* |
| GSE34496 | 0.954±0.108 | 0.929±0.136 | 0.923±0.120* | 0.797±0.199* | **0.965±0.084** | 0.851±0.179* | 0.910±0.144* | 0.920±0.132* | 0.958±0.102 |
| GSE35834-1 | 0.990±0.057 | 0.984±0.070 | **0.993±0.047** | 0.881±0.175* | 0.984±0.070 | 0.902±0.159* | 0.968±0.119 | 0.964±0.103* | 0.978±0.083 |
| GSE35834-2 | **1.000±0.000** | 0.980±0.090* | 0.985±0.075* | 0.868±0.208* | 0.967±0.116* | 0.932±0.156* | 0.982±0.082* | 0.975±0.093* | **1.000±0.000** |
| GSE35834-3 | 0.918±0.168 | 0.905±0.174 | 0.835±0.210* | 0.848±0.197* | **0.930±0.159** | 0.843±0.208* | 0.905±0.179 | 0.845±0.214* | 0.877±0.195* |
| GSE41282 | **0.990±0.070** | 0.980±0.098 | 0.955±0.144* | 0.895±0.205* | 0.920±0.184* | 0.910±0.193* | 0.965±0.128 | 0.940±0.163* | 0.920±0.184* |
| GSE108153 | **1.000±0.000** | 0.997±0.033 | 0.995±0.050 | 0.972±0.114* | 0.988±0.083 | 0.940±0.153* | 0.985±0.086 | 0.995±0.050 | 0.997±0.033 |
| Average | **0.948±0.110** | 0.924±0.137 | 0.923±0.137 | 0.873±0.184 | 0.903±0.149 | 0.878±0.184 | 0.909±0.159 | 0.933±0.130 | 0.921±0.135 |
| Win/Tie/Loss | # | 6/10/1 | 8/9/0 | 12/5/0 | 7/9/1 | 15/2/0 | 7/10/0 | 6/10/1 | 6/11/0 |

Table S2. Comparison on the miRNA datasets in specificity.

| Datasets | miRMarker | SVM-RFE | WGCNA | DNB | GroupBN | NGTM | QLCD | N-DAG | DDRM |
| --- | --- | --- | --- | --- | --- | --- | --- | --- | --- |
| GSE31164-1 | **0.853±0.191** | 0.823±0.186 | 0.847±0.186 | 0.813±0.203 | 0.753±0.147* | 0.800±0.195* | 0.840±0.180 | 0.837±0.192 | 0.833±0.180 |
| GSE31164-2 | **0.920±0.151** | 0.860±0.191* | 0.910±0.163 | 0.787±0.209* | 0.717±0.160* | 0.813±0.219* | 0.817±0.219* | 0.880±0.193 | 0.880±0.193 |
| GSE31164-3 | 0.758±0.194 | 0.710±0.199 | 0.727±0.218 | 0.725±0.207 | **0.827±0.040*** | 0.738±0.219 | 0.718±0.217 | 0.680±0.209* | 0.692±0.192* |
| GSE39046-1 | 0.960±0.109 | 0.953±0.116 | 0.933±0.134 | 0.767±0.187* | 0.930±0.136 | 0.857±0.172* | 0.930±0.136 | **0.977±0.085** | 0.963±0.105 |
| GSE39046-2 | **0.973±0.091** | 0.927±0.139* | 0.963±0.105 | 0.863±0.190* | 0.760±0.260* | 0.837±0.192* | 0.847±0.186* | 0.967±0.101 | 0.963±0.105 |
| GSE39046-3 | **0.968±0.102** | 0.915±0.173* | 0.963±0.105 | 0.887±0.183* | 0.885±0.185* | 0.868±0.193* | 0.870±0.186* | 0.942±0.137 | 0.927±0.150* |
| GSE41574 | 0.970±0.119 | 0.965±0.128 | 0.980±0.098 | 0.915±0.189* | 0.835±0.236* | 0.905±0.197* | 0.945±0.157 | 0.955±0.144 | **1.000±0.000*** |
| GSE67139 | 0.916±0.115 | 0.869±0.156* | 0.879±0.136* | 0.811±0.189* | 0.874±0.146* | 0.862±0.152* | 0.911±0.127 | **0.929±0.106** | 0.896±0.128 |
| GSE32273-1 | 0.930±0.169 | 0.948±0.151 | 0.925±0.175 | 0.923±0.178 | **0.952±0.147** | 0.818±0.235* | 0.947±0.148 | 0.928±0.168 | 0.927±0.176 |
| GSE32273-2 | 0.813±0.230 | 0.802±0.240 | **0.925±0.178*** | 0.815±0.232 | 0.888±0.202* | 0.783±0.243 | 0.777±0.250 | 0.793±0.245 | 0.843±0.233 |
| GSE32273-3 | **0.847±0.223** | 0.833±0.232 | 0.828±0.225 | 0.815±0.247 | 0.732±0.248* | 0.793±0.238 | 0.798±0.249 | 0.812±0.228 | 0.818±0.229 |
| GSE34496 | 0.987±0.081 | 0.968±0.110 | 0.963±0.113* | 0.903±0.178* | **0.997±0.033** | 0.953±0.123* | 0.970±0.112 | 0.985±0.075 | **0.997±0.033** |
| GSE35834-1 | **0.993±0.047** | 0.990±0.057 | 0.990±0.057 | 0.878±0.192* | 0.985±0.075 | 0.910±0.170* | 0.973±0.100 | 0.973±0.100 | **0.993±0.047** |
| GSE35834-2 | **1.000±0.000** | 0.973±0.108* | 0.988±0.068 | 0.848±0.217* | **1.000±0.000** | 0.937±0.155* | 0.992±0.060 | 0.943±0.150* | 0.987±0.077 |
| GSE35834-3 | **0.906±0.149** | 0.839±0.183* | 0.821±0.203* | 0.789±0.204* | 0.764±0.177* | 0.796±0.200* | 0.873±0.179 | 0.833±0.186* | 0.843±0.182* |
| GSE41282 | 0.900±0.201 | **0.950±0.151*** | 0.885±0.211 | 0.765±0.251* | 0.910±0.193 | 0.805±0.245* | 0.905±0.197 | 0.870±0.220 | 0.890±0.208 |
| GSE108153 | 0.970±0.119 | 0.910±0.193* | **0.980±0.098** | 0.897±0.203* | 0.880±0.211* | 0.852±0.225* | 0.925±0.179* | 0.962±0.132 | 0.962±0.132 |
| Average | **0.922±0.135** | 0.896±0.160 | 0.912±0.145 | 0.835±0.203 | 0.864±0.153 | 0.843±0.198 | 0.885±0.170 | 0.898±0.157 | 0.907±0.139 |
| Win/Tie/Loss | # | 7/9/1 | 3/13/1 | 12/5/0 | 9/6/2 | 14/3/0 | 4/13/0 | 3/14/0 | 3/13/1 |

Table S3. Comparison on the miRNA datasets in MCC.

| Datasets | miRMarker | SVM-RFE | WGCNA | DNB | GroupBN | NGTM | QLCD | N-DAG | DDRM |
| --- | --- | --- | --- | --- | --- | --- | --- | --- | --- |
| GSE31164-1 | 0.071±0.260 | **0.260±0.359*** | 0.032±0.307 | -0.016±0.130* | -0.039±0.120* | -0.006±0.234* | 0.141±0.314 | 0.058±0.224 | 0.137±0.326 |
| GSE31164-2 | **0.637±0.400** | 0.487±0.406* | 0.629±0.420 | -0.010±0.189* | 0.109±0.286* | 0.106±0.374* | 0.331±0.465* | 0.511±0.375* | 0.509±0.410* |
| GSE31164-3 | -0.002±0.079 | **0.188±0.384*** | 0.004±0.188 | -0.004±0.031 | -0.002±0.037 | -0.006±0.071 | -0.020±0.063 | -0.010±0.050 | -0.021±0.135 |
| GSE39046-1 | 0.835±0.241 | 0.743±0.260* | 0.783±0.249 | -0.012±0.385* | 0.792±0.250* | 0.519±0.390* | 0.761±0.278* | **0.840±0.191** | 0.822±0.226 |
| GSE39046-2 | 0.843±0.250 | 0.722±0.299* | 0.791±0.282 | 0.432±0.391* | 0.332±0.373* | 0.350±0.442* | 0.563±0.346* | 0.834±0.277 | **0.847±0.238** |
| GSE39046-3 | 0.815±0.270 | 0.813±0.299 | **0.822±0.261** | 0.495±0.452* | 0.607±0.356* | 0.441±0.408* | 0.436±0.440* | 0.720±0.287* | 0.737±0.316* |
| GSE41574 | 0.815±0.277 | 0.762±0.337* | 0.695±0.386* | 0.225±0.450* | 0.272±0.363* | 0.336±0.465* | 0.679±0.368* | 0.777±0.315 | **0.865±0.250** |
| GSE67139 | 0.691±0.206 | 0.579±0.229* | 0.624±0.234* | 0.432±0.340* | 0.610±0.246* | 0.552±0.223* | 0.638±0.211* | **0.697±0.198** | 0.626±0.215* |
| GSE32273-1 | 0.733±0.329 | 0.632±0.348* | 0.715±0.329 | 0.701±0.363 | **0.751±0.323** | 0.331±0.493* | 0.682±0.361 | 0.695±0.336 | 0.685±0.350 |
| GSE32273-2 | 0.386±0.428 | 0.213±0.520* | **0.681±0.373*** | 0.308±0.456 | 0.595±0.387* | 0.223±0.510* | 0.135±0.500* | 0.415±0.448 | 0.197±0.502* |
| GSE32273-3 | **0.504±0.435** | 0.356±0.467* | 0.269±0.509* | 0.377±0.496* | 0.100±0.333* | 0.094±0.527* | 0.155±0.565* | 0.464±0.401 | 0.248±0.426* |
| GSE34496 | 0.809±0.231 | 0.757±0.249 | 0.734±0.279* | 0.186±0.354* | **0.853±0.210** | 0.499±0.391* | 0.745±0.265* | 0.651±0.331* | 0.808±0.228 |
| GSE35834-1 | 0.894±0.187 | 0.902±0.184 | **0.929±0.147** | 0.401±0.412* | 0.903±0.186 | 0.572±0.422* | 0.833±0.236* | 0.791±0.277* | 0.910±0.182 |
| GSE35834-2 | **0.965±0.111** | 0.874±0.212* | 0.955±0.123 | 0.375±0.422* | 0.959±0.117 | 0.660±0.411* | 0.903±0.203* | 0.810±0.277* | 0.955±0.130 |
| GSE35834-3 | **0.629±0.330** | 0.488±0.365* | 0.270±0.416* | 0.077±0.359* | 0.275±0.411* | 0.088±0.427* | 0.611±0.363 | 0.297±0.394* | 0.458±0.404* |
| GSE41282 | 0.734±0.337 | **0.763±0.308** | 0.607±0.403* | 0.073±0.489* | 0.561±0.423* | 0.272±0.545* | 0.654±0.455 | 0.425±0.498* | 0.503±0.412* |
| GSE108153 | **0.889±0.226** | 0.770±0.356* | 0.829±0.265 | 0.658±0.392* | 0.684±0.382* | 0.539±0.484* | 0.769±0.324* | 0.853±0.260 | 0.796±0.349* |
| Average | **0.662±0.270** | 0.606±0.328 | 0.610±0.304 | 0.276±0.359 | 0.492±0.283 | 0.328±0.401 | 0.530±0.339 | 0.578±0.302 | 0.593±0.300 |
| Win/Tie/Loss | # | 11/4/2 | 6/10/1 | 14/3/0 | 11/5/1 | 16/1/0 | 12/5/0 | 7/10/0 | 8/9/0 |

Table S4. Comparison on the multi-class datasets.

| Metrics | Datasets | miRMarker | SVM-RFE | WGCNA | DNB | GroupBN | NGTM | QLCD | N-DAG | DDRM |
| --- | --- | --- | --- | --- | --- | --- | --- | --- | --- | --- |
| AUC | GSE31164 | **0.723±0.076** | 0.711±0.098 | 0.696±0.081* | 0.648±0.065* | 0.640±0.036* | 0.664±0.089* | 0.671±0.084* | 0.716±0.071 | 0.690±0.075* |
|  | GSE39046 | **0.903±0.063** | 0.885±0.077* | 0.887±0.076* | 0.772±0.083* | 0.815±0.088* | 0.744±0.107* | 0.795±0.096* | **0.903±0.060** | 0.881±0.084* |
|  | GSE35834 | 0.865±0.077 | **0.878±0.080** | 0.838±0.090* | 0.698±0.087* | 0.812±0.070* | 0.713±0.092* | 0.816±0.088* | 0.856±0.082 | 0.851±0.080 |
|  | Average | **0.830±0.072** | 0.825±0.085 | 0.807±0.083 | 0.706±0.078 | 0.756±0.064 | 0.707±0.096 | 0.761±0.089 | 0.825±0.071 | 0.807±0.080 |
| Sensitivity | GSE31164 | **0.763±0.138** | 0.733±0.179 | 0.741±0.136 | 0.662±0.189* | 0.647±0.075* | 0.707±0.191* | 0.744±0.152 | 0.755±0.138 | 0.714±0.163* |
|  | GSE39046 | **0.915±0.102** | 0.877±0.138* | 0.897±0.103 | 0.766±0.140* | 0.810±0.138* | 0.767±0.170* | 0.801±0.135* | 0.908±0.102 | 0.881±0.124* |
|  | GSE35834 | 0.864±0.114 | **0.877±0.110** | 0.824±0.133* | 0.720±0.182* | 0.792±0.126* | 0.755±0.155* | 0.821±0.133* | 0.842±0.119 | 0.831±0.128* |
|  | Average | **0.847±0.118** | 0.829±0.142 | 0.821±0.124 | 0.716±0.170 | 0.750±0.113 | 0.743±0.172 | 0.789±0.140 | 0.835±0.119 | 0.809±0.138 |
| Specificity | GSE31164 | 0.627±0.136 | **0.638±0.169** | 0.605±0.140 | 0.622±0.167 | 0.612±0.059 | 0.595±0.162 | 0.559±0.158* | 0.623±0.141 | 0.631±0.149 |
|  | GSE39046 | 0.770±0.064 | 0.771±0.077 | 0.754±0.096 | 0.674±0.143* | 0.695±0.140* | 0.638±0.166* | 0.675±0.151* | **0.773±0.069** | 0.761±0.088 |
|  | GSE35834 | 0.724±0.131 | **0.758±0.096*** | 0.726±0.124 | 0.615±0.175* | 0.717±0.133 | 0.593±0.151* | 0.681±0.151* | 0.739±0.117 | 0.732±0.112 |
|  | Average | 0.707±0.110 | **0.722±0.114** | 0.695±0.120 | 0.637±0.162 | 0.674±0.111 | 0.609±0.160 | 0.638±0.153 | 0.712±0.109 | 0.708±0.116 |
| MCC | GSE31164 | 0.044±0.192 | **0.223±0.258*** | -0.003±0.215 | -0.017±0.087* | -0.007±0.054* | 0.001±0.103* | 0.043±0.188 | 0.008±0.157 | 0.009±0.204 |
|  | GSE39046 | **0.761±0.183** | 0.734±0.187 | 0.716±0.219 | 0.580±0.237* | 0.564±0.219* | 0.329±0.305* | 0.485±0.222* | 0.755±0.178 | 0.701±0.203* |
|  | GSE35834 | 0.676±0.224 | **0.720±0.193** | 0.599±0.258* | 0.082±0.259* | 0.642±0.210 | 0.157±0.303* | 0.574±0.233* | 0.642±0.225 | 0.629±0.222 |
|  | Average | 0.494±0.199 | **0.559±0.213** | 0.437±0.231 | 0.215±0.194 | 0.400±0.161 | 0.162±0.237 | 0.367±0.214 | 0.468±0.187 | 0.446±0.210 |

Table S5. Details of the colorectal cancer independent validation datasets.

| Datasets | Sources | # of Normal Samples | # of Colorectal Cancer Samples |
| --- | --- | --- | --- |
| Validation set 1 | GSE112264 | 41 | 50 |
| Validation set 2 | GSE113486 | 100 | 40 |
| Validation set 3 | GSE211692 | 5643 | 1596 |

Note: Mark “#” indicates the quantity.


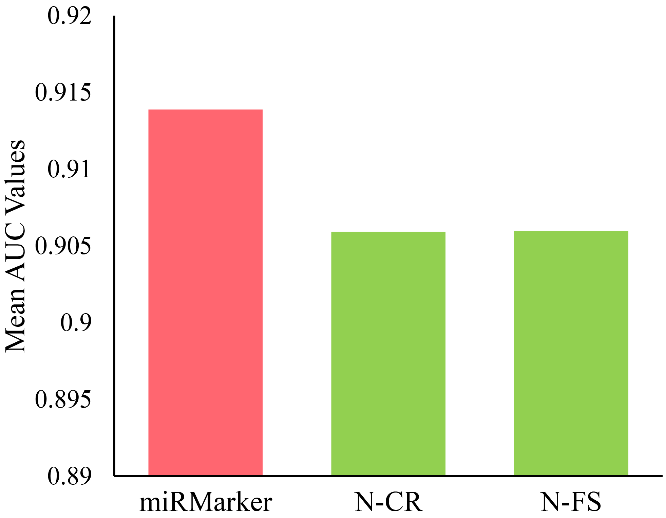


Fig. S1. Comparison with N-CR and N-FS in average AUC.


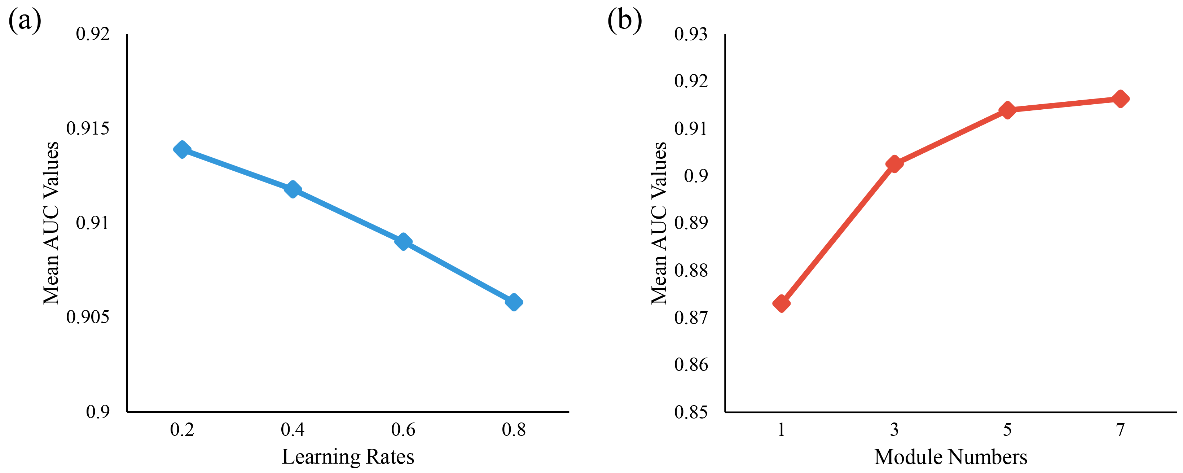


Fig. S2. Parameter sensitivity analysis. (a) learning rate *α*; (b) module number *g*.

**Module-Disease Network Analysis for Colorectal Cancer**

We applied miRMarker to define the potential miRNA biomarkers for colorectal cancer diagnosis on a real-world transcriptomics dataset from GEO database (GSE108153). Five miRNA modules were identified (Fig. 3(a)-(e) in the manuscript).

To explore the relationships between the defined miRNA modules and colorectal cancer, we constructed the module-disease network (Fig. 5 in the manuscript) according to the experimentally verified miRNA-disease relations in miRCancer and miR2Disease. The module-disease network contains two types of nodes: module nodes (pink circles) and disease nodes (blue diamonds). If one disease was associated with any miRNA in a module, we built an edge (undirected, light line) between the disease node and module node. If there is an inclusion relationship between two diseases in MeSH, we linked the father disease node to child disease node with a directed edge (dark line).

In the module-disease network, one module node is connected to many disease nodes. It is noteworthy that all the five module nodes are connected to the disease node of colorectal neoplasms (darkest blue), reflecting that the defined modules are closely related to the occurrence and development of colorectal neoplasms. miRMarker really identified the colorectal cancer-related miRNA modules.

Moreover, there are ten other disease nodes (darkest blue) linked to all the five module nodes. The eleven co-connected disease nodes represent the different neoplastic diseases, demonstrating the complexity of regulatory mechanisms of miRNAs on cancers. A specific miRNA module may affect multiple cancers. Meanwhile, one cancer is correlated with many miRNA modules. The node “Neoplasms” points to most disease nodes, forming a large neoplastic disease module, which also shows the important place of miRNAs in cancer study.

**Gene Expression Analysis in Colorectal Cancer-Related Pathways**

The pathway analysis was conducted for the target genes of the five hub miRNAs (hsa-miR-135b-5p, hsa-miR-224-5p, hsa-miR-183-5p, hsa-miR-96-5p and hsa-miR-195-5p) using the online tool DAVID. Typically, fifty-one target genes were enriched in the KEGG pathway “Colorectal cancer” (hsa05210) with a false discovery rate (FDR) of 2.55e-06. To further understand the mechanism of miRNA regulating genes in colorectal cancer development, we performed the gene expression analysis using The Cancer Genome Atlas (TCGA) Colon Cancer cohort. The TCGA gene expression data of colon cancer was derived from the UCSC Xena platform (http://xena.ucsc.edu/). Forty-one normal samples and 453 primary colon tumor samples were included in the gene expression analysis. Fig. S3 shows the overview of dysregulated pathway “Colorectal cancer” (hsa05210). The genes significantly upregulated and downregulated in the cancer samples are colored with red and green respectively.

The gene dysregulation in colorectal cancer involves the important cellular function pathways “Cell cycle” (hsa04110, enrichment FDR = 5.01e-12), “Apoptosis” (hsa04210, enrichment FDR = 3.75e-03), and several critical signaling pathways Wnt signaling pathway (hsa04310, enrichment FDR = 2.20e-04), PI3K-Akt signaling pathway (hsa04151, enrichment FDR = 3.78e-05), TGF-*β* signaling pathway (hsa04350, enrichment FDR = 1.64e-04), MAPK signaling pathway (hsa04010, enrichment FDR = 3.78e-05), p53 signaling pathway (hsa04115, enrichment FDR = 2.36e-05), mTOR signaling pathway (hsa04150, enrichment FDR = 1.74e-04), and ErbB signaling pathway (hsa04012, enrichment FDR = 1.43e-03). Table S6 gives the regulatory relationships between the hub miRNAs and the enriched target genes in the pathway “Colorectal cancer” (hsa05210). One target gene is usually regulated by several hub miRNAs. Besides, the target genes of one hub miRNA distributed in multiple pathways related to colorectal cancer. The dysregulation of one hub miRNA can cause the disturbance of multiple colorectal cancer-related pathways, promoting the development of colorectal cancer further jointly.

Wnt signaling pathway is the main dysregulated pathway in colorectal cancer. The slight disturbance in this pathway can cause the formation of cancer cells [1]. In Wnt signaling pathway, *β*-catenin accumulates in the cytoplasm then enters the nucleus to combine with T-cell factors (TCF), promoting the cancer development. We observed the significant upregulation of the oncogene catenin beta 1 (CTNNB1, *β*-catenin), indicating Wnt signaling pathway has been activated. The level of tumor suppressor APC regulator of WNT signaling pathway (APC) decreases greatly in cancer group, regulated by the hub miRNAs hsa-miR-183-5p and hsa-miR-224-5p. The overexpression of these two miRNAs may be one of the main factors causing the great disturbance in Wnt signaling pathway to move forward the colorectal cancer process. MAPK signaling pathway is one of the major pathways controlling the cell proliferation. Its activation of signaling cascades is closely associated with cancer pathogenesis [2]. We noticed the downregulation of oncogene KRAS proto-oncogene, GTPase (KRAS) and several key kinases including the extracellular-signal-regulated kinases (ERK, RAF1/BRAF/MAP2K1/MAP2K2/MAPK1/MAPK3) and c-Jun N-terminal or stress-activated protein kinases (JNK, MAPK8/MAPK9/MAPK10). Whereas, the downstream transcription factors Jun proto-oncogene, AP-1 transcription factor subunit (JUN), MYC proto-oncogene, bHLH transcription factor (MYC) and cyclin D1 (CCDN1) overexpress in cancer group, indicating their important roles in the differentiation and proliferation of colorectal cancer cells. The regulatory effects of oncogenes MYC and JUN in cancer metabolism reprogramming have been discussed in previous studies [3, 4]. The downregulation of hsa-miR-195-5p in colorectal cancer samples may result in the overexpression of MYC and JUN. There is complex crosstalk between mTOR signaling pathway, PI3K-Akt signaling pathway and MAPK signaling pathway. In normal state, mTOR signaling pathway and PI3K-Akt signaling pathway regulate various cellular processes, such as cell growth and differentiation. In tumors, mTOR/PI3K-Akt signaling pathways are activated for satisfying the needs of rapid growth, proliferation and metastasis of tumor cells [5, 6]. In Fig. S3, the downregulation of multiple kinases in PI3K-Akt/mTOR signaling pathways may represent the suppression of tumors by the immune system in early cancer stage. The epidermal growth factor (EGF) and its receptor (EGFR) in ErbB signaling pathway also downregulated, which controls the downstream signal transduction in mTOR signaling pathway. TGF-*β* signaling pathway plays a dual role in tumor development [7]. In early tumors, TGF-*β* restrains cancer by inducing the apoptosis of tumor cells. However, with the development of cancer, TGF-*β* promotes tumors by numerous regulatory mechanisms. In this study, there is no significant change in the expression levels of TGFB2 and TGFB3 between different sample groups. But the TGF-*β* receptor TGFBR1 overexpresses in the cancer group. The downstream tumor suppressors SMAD family members (SMAD2/SMAD3/SMAD4) are downregulated significantly, meaning TGF-*β* signaling pathway is suppressed. hsa-miR-224-5p and hsa-miR-183-5p may be the main factors in the inhibition of TGF-*β* signaling pathway. The transcription factor p53 is one of the key tumor suppressors by performing a variety of cellular activities, such as DNA repair and cell apoptosis [8]. In normal cells, p53 maintains low expression level to prevent the inappropriate cellular responses. Nevertheless, p53 mutations widely exist in colorectal tumors. The overexpression of p53 mutant is tightly associated with the occurrence and development of colorectal cancer [9]. We observed the significant upregulation of p53 in colorectal cancer tissues and the downregulation of its two downstream effectors cyclin dependent kinase inhibitor 1A (CDKN1A, p21) and BCL2 antagonist/killer 1 (BAK1). Actually, CDKN1A can be a tumor suppressor or an oncogene, mainly determined by the tumor microenvironment [10]. The downregulation of BAK1 in colorectal cancer tissues was also found in precious study [11].


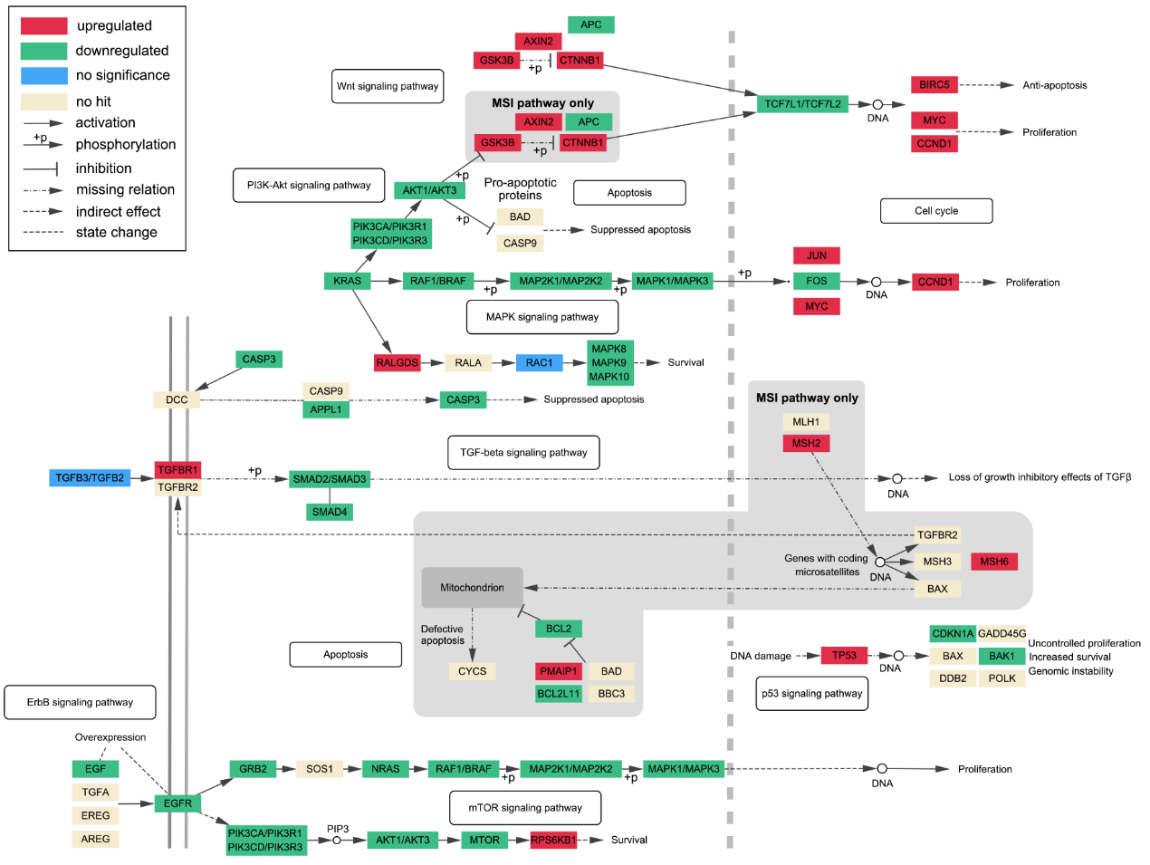


Fig. S3. Overview of the dysregulated KEGG pathway “Colorectal cancer” (hsa05210).

Table S6. Regulation relationships between hub miRNAs and 51 targets enriched in pathway “Colorectal cancer” (hsa05210).

| Ensembl ID | Symbols | hsa-miR-135b-5p | hsa-miR-224-5p | hsa-miR-183-5p | hsa-miR-96-5p | hsa-miR-195-5p |
| --- | --- | --- | --- | --- | --- | --- |
| ENSG00000177885 | GRB2 |  |  |  |  | √ |
| ENSG00000175387 | SMAD2 |  |  |  |  | √ |
| ENSG00000166949 | SMAD3 |  |  |  |  | √ |
| ENSG00000133703 | KRAS |  | √ | √ | √ |  |
| ENSG00000177606 | JUN |  | √ |  |  | √ |
| ENSG00000082701 | GSK3B |  | √ | √ | √ | √ |
| ENSG00000138798 | EGF |  |  |  |  | √ |
| ENSG00000030110 | BAK1 |  | √ |  |  |  |
| ENSG00000126934 | MAP2K2 |  | √ |  |  |  |
| ENSG00000102882 | MAPK3 |  |  |  |  | √ |
| ENSG00000107643 | MAPK8 |  | √ |  | √ | √ |
| ENSG00000106799 | TGFBR1 | √ |  |  | √ | √ |
| ENSG00000171791 | BCL2 |  | √ |  | √ | √ |
| ENSG00000169032 | MAP2K1 |  |  |  |  | √ |
| ENSG00000170345 | FOS |  |  | √ |  |  |
| ENSG00000157500 | APPL1 |  | √ |  | √ |  |
| ENSG00000119699 | TGFB3 |  | √ |  |  |  |
| ENSG00000110092 | CCND1 |  | √ | √ | √ | √ |
| ENSG00000141682 | PMAIP1 |  | √ | √ | √ | √ |
| ENSG00000164305 | CASP3 |  | √ |  |  |  |
| ENSG00000141646 | SMAD4 |  | √ | √ |  |  |
| ENSG00000124762 | CDKN1A |  | √ |  | √ | √ |
| ENSG00000109339 | MAPK10 |  |  |  |  | √ |
| ENSG00000095002 | MSH2 |  |  | √ |  |  |
| ENSG00000152284 | TCF7L1 |  | √ |  |  |  |
| ENSG00000136997 | MYC | √ |  | √ |  | √ |
| ENSG00000132155 | RAF1 |  |  |  |  | √ |
| ENSG00000136238 | RAC1 |  | √ | √ |  |  |
| ENSG00000146648 | EGFR |  | √ |  |  |  |
| ENSG00000168036 | CTNNB1 |  |  |  |  | √ |
| ENSG00000116062 | MSH6 |  |  | √ |  |  |
| ENSG00000160271 | RALGDS |  |  | √ |  |  |
| ENSG00000213281 | NRAS |  | √ | √ |  |  |
| ENSG00000153094 | BCL2L11 |  | √ | √ | √ | √ |
| ENSG00000142208 | AKT1 | √ |  |  |  |  |
| ENSG00000089685 | BIRC5 | √ |  |  |  | √ |
| ENSG00000145675 | PIK3R1 |  | √ |  |  | √ |
| ENSG00000141510 | TP53 |  |  | √ |  |  |
| ENSG00000092969 | TGFB2 |  |  |  |  | √ |
| ENSG00000108443 | RPS6KB1 |  |  |  |  | √ |
| ENSG00000100030 | MAPK1 |  |  | √ |  |  |
| ENSG00000050748 | MAPK9 |  |  |  |  | √ |
| ENSG00000198793 | MTOR |  | √ |  |  |  |
| ENSG00000157764 | BRAF |  |  | √ |  |  |
| ENSG00000134982 | APC | √ | √ |  |  | √ |
| ENSG00000148737 | TCF7L2 |  | √ | √ | √ |  |
| ENSG00000117461 | PIK3R3 |  | √ |  |  |  |
| ENSG00000117020 | AKT3 |  |  |  |  | √ |
| ENSG00000121879 | PIK3CA |  |  |  |  | √ |
| ENSG00000171608 | PIK3CD |  |  |  |  | √ |
| ENSG00000168646 | AXIN2 |  |  |  |  | √ |

Note: “√” indicates the gene (row) is the target of miRNA (column).

**External Validation Experiments on Three Different Diseases**

We conducted three supplementary experiments with external validation to verify the effectiveness of miRMarker for potential biomarker identification of different diseases.

Three challenging problems were included: the prognosis of nasopharyngeal carcinoma (NPC), the diagnosis of recurrent implantation failure (RIF) and the severity judgement of COVID-19. NPC is a malignant tumor distributed in southern China and Southeast Asia. Although NPC tumor could be controlled locally through radiotherapy, its high recurrence and distant metastasis remains a key challenge [12]. A common definition of RIF is the pregnancy failure after transferring more than 3 good-quality blastocysts [13]. About fifty percent RIF patients fail to identify the causes, posing the enormous and urgent challenges for clinicians [14]. COVID-19 brings the huge loss of life and property for the people throughout the world in the past years. Although most countries have lifted the restrictions on COVID-19, its threat will persist for a long time. Focusing on the severe COVID-19 patients has become the critical point for preventing the COVID-19 variants [15].

Table S7 gives the details of the involved discovery sets and external validation sets. These datasets were preprocessed using the same manner as the datasets in the manuscript including the miRNA names mapping. For NPC experiment, the discovery set contains 39 NPC samples developed with recurrent disease and 86 samples without recurrent disease. Another group of NPC samples, consisting of 48 samples with recurrent disease and 73 samples without recurrent disease, was included as the external validation set. For RIF experiment, a sample group containing 80 control samples and 31 case samples was used as the discovery set. Another sample group was used as the external validation set, containing 62 control samples and 29 case samples. For COVID-19 experiment, we focused on the discrimination between the non-severe group and severe group. The discovery set contains 66 moderate severe samples and 83 severe samples. Another group was included as the external validation set, which consists of 18 non-severe samples (mild severe) and 18 severe samples.

As performed for colorectal cancer in the manuscript, the identified potential biomarkers by miRMarker on the discovery set were validated on the corresponding external validation set. Table S8 shows the results of the three supplementary experiments. For distinguishing different sample groups, miRMarker obtained the AUC values of 0.922 for NPC, 0.989 for RIF, 0.895 for COVID-19 on the discovery sets, respectively. On the three external validation sets, the AUC values are 0.731 for NPC, 0.967 for RIF and 1.000 for COVID-19, respectively. miRMarker performed well in the experiments of NPC, RIF and COVID-19. The results of supplementary experiments further illustrate the effectiveness of miRMarker in identifying the potential biomarkers for diseases.

Table S7. Details of the datasets in three supplementary experiments.

| Datasets | Involved Diseases | # of Features  (Discovery set) | # of Samples  (Discovery set) | # of Samples  (External validation set) | Sources |
| --- | --- | --- | --- | --- | --- |
| NPC | Nasopharyngeal Carcinoma | 149 | 125 (86:39) | 121 (73:48) | [16] |
| RIF | Recurrent Implantation Failure | 473 | 111 (80:31) | 91 (62:29) | [17] |
| COVID-19 | COVID-19 | 686 | 149 (66:83) | 36 (18:18) | [18, 19] |

Note: Mark “#” indicates the quantity.

Table S8. Performance of miRMarker in three supplementary experiments.

| Datasets | AUC values  (Discovery set) | AUC values  (External validation set) |
| --- | --- | --- |
| NPC | 0.922 | 0.731 |
| RIF | 0.989 | 0.967 |
| COVID-19 | 0.895 | 1.000 |

**References**

[1] Jafarzadeh M, Soltani BM. MiRNA-Wnt signaling regulatory network in colorectal cancer. *J Biochem Mol Toxicol* 2021;35(10):e22883.

[2] Fang JY, Richardson BC. The MAPK signalling pathways and colorectal cancer. *Lancet Oncol* 2005;6(5):322-7.

[3] Satoh K, Yachida S, Sugimoto M, et al. Global metabolic reprogramming of colorectal cancer occurs at adenoma stage and is induced by MYC. *Proc Natl Acad Sci* 2017;114(37):E7697-706.

[4] Khalid AQ, Bhuvanendran S, Magalingam KB, et al. Clinically relevant genes and proteins modulated by tocotrienols in human colon cancer cell lines: systematic scoping review. *Nutrients* 2021;13(11):4056.

[5] Zou Z, Tao T, Li H, et al. mTOR signaling pathway and mTOR inhibitors in cancer: progress and challenges. *Cell Biosci* 2020;10:31.

[6] Koveitypour Z, Panahi F, Vakilian M, et al. Signaling pathways involved in colorectal cancer progression. *Cell Biosci* 2019;9:97.

[7] Brown NF, Marshall JF. Integrin-Mediated TGFβ Activation Modulates the Tumour Microenvironment. *Cancers* 2019;11(9):1221.

[8] Liebl MC, Hofmann TG. The role of p53 signaling in colorectal cancer. *Cancers* 2021;13(9):2125.

[9] Borrero LJH, El-Deiry WS. Tumor suppressor p53: biology, signaling pathways, and therapeutic targeting. *Biochim Biophys Acta-Rev Cancer* 2021;1876(1):188556.

[10] Kreis NN, Louwen F, Yuan J. The Multifaceted p21 (Cip1/Waf1/CDKN1A) in Cell Differentiation, Migration and Cancer Therapy. *Cancers* 2019;11(9):1220.

[11] Liu C, Zhang A, Cheng L, et al. miR‑410 regulates apoptosis by targeting Bak1 in human colorectal cancer cells. *Mol Med Rep* 2016;14(1):467-73.

[12] Lee AWM, Ma BBY, Ng WT, et al. Management of nasopharyngeal carcinoma: current practice and future perspective. *J Clin Oncol* 2015;33(29):3356-64.

[13] Shaulov T, Sierra S, Sylvestre C. Recurrent implantation failure in IVF: A Canadian Fertility and Andrology Society Clinical Practice Guideline. *Reprod Biomed Online* 2020;41(5):819-33.

[14] Bashiri A, Halper KI, Orvieto R. Recurrent implantation failure-update overview on etiology, diagnosis, treatment and future directions. *Reprod Biol Endocrinol* 2018;16:121.

[15] Davis HE, McCorkell L, Vogel JM, et al. Long COVID: major findings, mechanisms and recommendations. *Nat Rev Microbiol* 2023;21(3):133-46.

[16] Bruce JP, Hui AB, Shi W, et al. Identification of a microRNA signature associated with risk of distant metastasis in nasopharyngeal carcinoma. *Oncotarget* 2015;6(6):4537-50.

[17] Rekker K, Altmäe S, Suhorutshenko M, et al. A two-cohort RNA-seq study reveals changes in endometrial and blood miRNome in fertile and infertile women. *Genes (Basel)* 2018;9(12):574.

[18] Zeng Q, Qi X, Ma J, et al. Distinct miRNAs associated with various clinical presentations of SARS-CoV-2 infection. *iScience* 2022;25(5):104309.

[19] Gutmann C, Khamina K, Theofilatos K, et al. Association of cardiometabolic microRNAs with COVID-19 severity and mortality. *Cardiovasc Res* 2022;118(2):461-74.
