# Supplementary material for: Identifying the potential miRNA biomarkers based on multi-view networks and reinforcement learning for diseases
Source: Brief Bioinform. 2023 Nov 28;25(1):bbad427. doi: 10.1093/bib/bbad427 (PMC10753537; doi:10.1093/bib/bbad427)
Supplement: table_s4_discussion_bbad427 [file table_s4_discussion_bbad427.docx]

Table S4 gives the comparison results for multi-class classification. In AUC, miRMarker outperformed the WGCNA, DNB, GroupBN, NGTM and QLCD on all the three datasets. Compared with SVM-RFE, miRMarker defeated it on one dataset and tied with it on two datasets in AUC. miRMarker outperformed DDRM on two datasets in AUC. Moreover, miRMarker obtained the best average performance over datasets in AUC (0.830) and sensitivity (0.847). miRMarker got the second-highest average MCC (0.494) on the multi-class datasets, which is only lower than that of SVM-RFE (0.559). Although miRMarker did not achieve the best performance in specificity, considering the four metrics (AUC, sensitivity, specificity and MCC) together, miRMarker also showed more advantageous performance than other methods in multi-class classification.
